# Complete chloroplast genomes of *Cerastium alpinum*, *C. arcticum* and *C. nigrescens*: genome structures, comparative and phylogenetic analysis

**DOI:** 10.1038/s41598-023-46017-y

**Published:** 2023-10-31

**Authors:** Sylwia E. Milarska, Piotr Androsiuk, Łukasz Paukszto, Jan P. Jastrzębski, Mateusz Maździarz, Keith W. Larson, Irena Giełwanowska

**Affiliations:** 1 Department of Plant Physiology, Genetics and Biotechnology, Faculty of Biology and Biotechnology, University of Warmia and Mazury in Olsztyn, ul. M. Oczapowskiego 1A, 10-719 Olsztyn, Poland; 2 Department of Botany and Nature Protection, Faculty of Biology and Biotechnology, University of Warmia and Mazury in Olsztyn, Pl. Łódzki 1, 10-721 Olsztyn, Poland; 3 Climate Impacts Research Centre, Umeå University, 90187 Umeå, Sweden

**Keywords:** Genetics, Plant sciences

## Abstract

The genus *Cerastium* includes about 200 species that are mostly found in the temperate climates of the Northern Hemisphere. Here we report the complete chloroplast genomes of *Cerastium alpinum*, *C*. *arcticum* and *C*. *nigrescens*. The length of cp genomes ranged from 147,940 to 148,722 bp. Their quadripartite circular structure had the same gene organization and content, containing 79 protein-coding genes, 30 tRNA genes, and four rRNA genes. Repeat sequences varied from 16 to 23 per species, with palindromic repeats being the most frequent. The number of identified SSRs ranged from 20 to 23 per species and they were mainly composed of mononucleotide repeats containing A/T units. Based on Ka/Ks ratio values, most genes were subjected to purifying selection. The newly sequenced chloroplast genomes were characterized by a high frequency of RNA editing, including both C to U and U to C conversion. The phylogenetic relationships within the genus *Cerastium* and family Caryophyllaceae were reconstructed based on the sequences of 71 protein-coding genes. The topology of the phylogenetic tree was consistent with the systematic position of the studied species. All representatives of the genus *Cerastium* were gathered in a single clade with *C. glomeratum* sharing the least similarity with the others.

## Introduction

The genus *Cerastium* belonging to the Caryophyllaceae family contains over 200 species^1^ and consists of herbaceous plants, annuals, and perennials^2,3^ that occur mainly in the Northern Hemisphere. The genus is most common in temperate and cold regions, especially at high elevations, with Eurasia serving as its center of diversity. The majority of the representatives of the genus *Cerastium* have a limited range with only a few species characterized by a cosmopolitan distribution^3^. The current state of knowledge about the genetic diversity of the genus *Cerastium* is based on a rather limited number of studies. Genetic investigations employed analysis of isoenzymatic polymorphism^4,5^ and different molecular markers like RAPD and SCAR^6^, AFLP^7,8^ and iPBS^9^. Apart from traditional genetic diversity studies, there are also papers that focused on the role of hybridization and introgression events in the evolution of the genus *Cerastium*^10–12^. One example of the intricated systematics within the genus *Cerastium* is the *C. alpinum*–*C. arcticum* complex. Vast physical variation within that complex has resulted in the identification of numerous species, subspecies, and varieties within that group of plants^13–16^, among which *C. alpinum* L., *C. arcticum* Lange and *C. nigrescens* (H.C. Watson) can be found^17–19^. *C. alpinum* is an arctic-alpine species that occurs in the northern part of North America and Europe. Moreover, it is recorded in Europe on high mountain grasslands, mostly in the subalpine zone (from 1480 to 1680 m.a.s.l.), where it forms one-species aggregations^20,21^. *C. arcticum* appeared here as the most problematic component of the species group. Latest genetic and morphological analyses suggest that the species conventionally known as *C. arcticum* actually consists of two separate taxa: *C. arcticum* s. str. and *C. nigrescens*^10,18,19^. The first of them is restricted to arctic areas (the Canadian Arctic, Greenland, Svalbard, north-western arctic Russian islands), while the other is characteristic to fell regions (the British Isles, Fennoscandian mountains, Faeroe Islands, Iceland). Despite the intensive studies delimitation of these taxa is still problematic on a large geographic scale^11^. Consequently, a novel approach is needed to find a universal marker for taxon identification.

Due to the recent progress observed in molecular sciences, high-throughput genome sequencing technologies have become widely available and provide a relatively fast and inexpensive way of obtaining high-quality genomic data. In case of the plant genetics, chloroplast (cp) genomes became a source of data commonly used in comparative studies^22,23^, biotechnology^24^, species identification^25,26^ or in analyses addressing phylogenetic questions^27,28^. It was shown that the complete cp genome contains roughly equivalent amount of information as the *cox1* gene used in animals, so it has the potential to provide enough distinguishing differences that enable molecular identification of even closely related species^29^. Using the entire chloroplast genome as a super-barcode is a novel approach that could potentially address the limitations of conventional two-locus barcoding^30^. Traditional barcoding primarily relies on sequence variation within two regions of the chloroplast genome, *matK* and *rbcL*, which is not always sufficient for precise species delimitation. To date, there are only two publicly available chloroplast genome sequences for the genus *Cerastium*, i.e. complete cp genome sequence for *C. glomeratum* and partial genome sequence for *C. arvense* (NC_066897 and MH627219, respectively; NCBI). The available data revealed that the *Cerastium* chloroplast genome has conserved quadripartite structure with size and gene content typical for angiosperms.. Except for the above-mentioned *C. glomeratum*, there are 60 other species (representing the following genera: *Agrostemma*, *Arenaria*, *Colobanthus*, *Dianthus*, *Gymnocarpos*, *Gypsophila*, *Lychnis*, *Myosoton*, *Paronychia*, *Psammosilene*, *Pseudostellaria*, *Silene,* and *Stellaria*) for which complete plastome sequences are available in the NCBI database (accessed on March 24, 2023). Considering the fact, that the Caryophyllaceae family consists of 40 genera and includes about 12 500 species, the number of chloroplast genomes currently available for this group of plants should be treated as very low.

The complete chloroplast genomes of three *Cerastium* species (*C*. *alpinum*, *C*. *arcticum* and *C*. *nigrescens*) have been sequenced and annotated for the first time in this paper. The specific objectives of this study included: (1) determination of the size and structure of cp genomes for* C*. *alpinum*, *C*. *arcticum* and *C*. *nigrescens*, (2) identification of genomic repeats, including forward, reverse, palindromic and complementary sequences among *Cerastium* chloroplast genomes, (3) identification and characterization of simple sequence repeats (SSRs) in newly sequenced *Cerastium* plastomes, (4) analysis of the evolution and dynamics of chloroplast protein-coding sequences, (5) comparative study of all available *Cerastium* chloroplast genomes, and (6) reconstruction of the phylogenetic relationships within genus *Cerastium* and family Caryophyllaceae based on plastome sequences.

## Results

### Organization of chloroplast genomes

**Figure 1:**
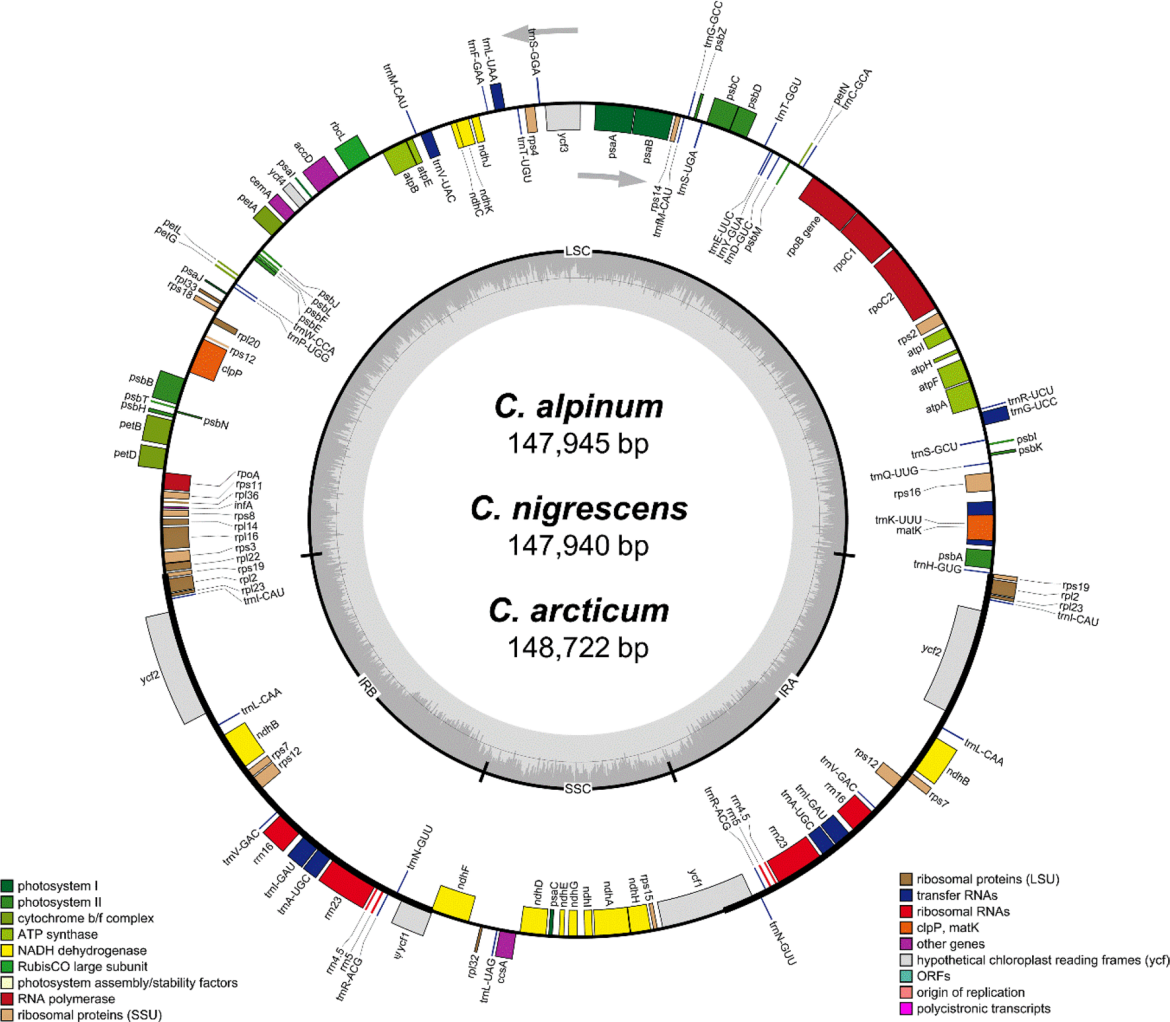
Gene map of the three *Cerastium* chloroplast genomes. Genes drawn inside the circle are transcribed clockwise, and those outside are transcribed counterclockwise (indicated by arrows). Differential functional gene groups are color-coded. GC content variations is shown in the middle circle.

NovaSeq Illumina platform was applied for chloroplast genome sequencing of three *Cerastium* species. The highest number of raw reads (15,370,470) was obtained for *C. arcticum*, whereas in the case of *C. nigrescens* and *C. alpinum* sequencing yielded 13,970,724 and 13,586,446 reads, respectively. The raw reads were then mapped separately to the reference chloroplast genome of *C*. *glomeratum*. As a result, 297,869 mapped reads with a mean coverage of 304 were observed for *C. nigrescens*, while in the case of two other species these value were more than twice as high and amounted to 650,075 reads and 664 coverage for *C. alpinum* and 664,454 reads and 675 coverage for *C. arcticum* (Supplementary FigureS1). The size of reported cp genomes was 147,945 for *C. alpinum*, 147,940 bp for *C*. *nigrescens* and 148,722 bp for *C*. *arcticum*. Each chloroplast genome appeared as a circular, double-stranded DNA molecule with a traditional quadripartite structure composed of Large Single Copy (LSC) and Small Single Copy (SSC) separated by a pair of Inverted Repeats (IR) regions which have identical sequences but opposite orientation (Fig. 1). The overall GC content was nearly identical in all *Cerastium* species: 36.51% for *C. alpinum*, 36.46% for *C. arcticum* and 36.52% for *C. nigrescens* (Table 1). Additionally, variant calling analysis revealed no heteroplasmy in reported chloroplast genomes.

**Table 1:** Summary of chloroplast genome characteristics of studied *Cerastium* species.

Genome features	*C. alpinum*	*C. arcticum*	*C. nigrescens*
Raw data reads no	13,586,446	15,370,470	13,970,724
Mapped reads no	650,075	664,454	297,869
Percent of chloroplast genome reads (%)	4.78	4.32	2.13
Mean coverage (x)	664	675	304
Size (bp)	147,945	148,722	147,940
LSC length (bp)	80,080	80,835	80,076
SSC length (bp)	16,851	16,861	16,850
IR length (bp)	25,507	25,513	25,507
Protein-coding genes	79	79	79
tRNA genes	30	30	30
rRNA genes	4	4	4
Number of genes duplicated in IR	18	18	18
Overall GC content (%)	36.51	36.46	36.52

All three reported *Cerastium* chloroplast genomes contained an identical set of 113 genes composed of 75 protein-coding genes, 30 transfer RNA genes, four ribosomal RNA genes, and four conserved chloroplast ORFs (*ycf1*, *ycf2*, *ycf3*, *ycf4*) (Table 2). We have also identified in each IR region a sequence for the *rpl23* gene which due to the internal, premature termination codon was retained rather as a nonfunctional pseudogene. Most protein-coding genes have the standard AUG as the initiation codon. The total number of codons for all protein-coding genes in the reported cp genomes was 26,115 for *C. arcticum*, 26,116 for *C. alpinum* and 26,220 for. *C. nigrescens*. All studied species shared similar pattern of codon usage and amino acid frequency. Leucine appeared as the dominant amino acid (10.7%), whereas cysteine was less frequently encountered (1.2%). The most abundant codon (4.46%) was ATT and the last (0.004%) were TTG (all species), ATA and CTG (*C*. *nigrescens*). CTG codon appeared only in *C*. *nigrescens* (Supplementary Table S1). Most of the genes in analyzed chloroplast genomes did not contain introns, 14 others contained one intron (*atpF*, *ndhA*, *ndhB*, *petB*, *petD*, *rpl16*, *rpoC1*, *rps16*, *trnA-UGC*, *trnG-UCC*, *trnI-GAU*, *trnK-UUU*, *trnL-UAA*, *trnV-UAC*), whereas only three genes consisted of three exons (*clpP*, *ycf3,* and *rps12*). Our data confirmed that the *rps12* gene, coding plastid ribosomal protein S12, is a trans-splicing gene. This gene was split into three exons: the first exon (5’end of the sequence) was located in the LSC, while the second and third exons in the IRs. The smallest intron was found in the *trnL-UAA* (518 bp for *C. arcticum* and 520 bp for *C. alpinum* and *C. nigrescens*), whereas the biggest was in the *trnK-UUU* (2479 bp for *C. arcticum* and 2480 bp for *C. alpinum* and *C. nigrescens*) gene. The *matK* gene was positioned inside the intron of *trnK*-UUU. Fifty-eight protein-coding genes, 22 tRNA genes, and two conserved chloroplast ORFs (*ycf3* and *ycf4*) were located in the LSC region, SSC region contained eleven protein-coding genes, one tRNA gene, and one chloroplast ORF (*ycf1*, located on the boundary between SSC and IR_B_), whereas repeated IR region contained six protein-coding genes (including *rps19* gene located on the boundary between IR_A_ and LSC), seven tRNA genes, four rRNA genes, and one chloroplast ORF (*ycf2*).

**Table 2:** List of genes present in chloroplast genome of *Cerastium.*

Category	Group of gene	Name of genes
Photosynthesis	Photosystem I	*psaA, psaB, psaC, psaI, psaJ*
	Photosystem II	*psbA, psbB, psbC, psbD, psbE, psbF, psbH, psbI, psbJ, psbK, psbL, psbM, psbN, psbT, psbZ*
	Cytochrome complex	*petA, petB, petD, petG, petL, petN*
	ATP synthase	*atpA, atpB, atpE, atpF, atpH, atpI*
	NADH dehydrogenase	*ndhA, ndhB* (× 2)*, ndhC, ndhD, ndhE, ndhF, ndhG, ndhH, ndhI, ndhJ, ndhK*
	Large subunit of RUBISCO	*rbcL*
DNA replication and protein synthesis	Ribosomal RNA	*rrn4.5* (× 2)*, rrn5* (× 2)*, rrn16* (× 2)*, rrn23* (× 2)
	Small subunit ribosomal proteins	*rps2, rps3, rps4, rps7* (× 2)*, rps8, rps11, rps12* (× 2)*, rps14, rps15, rps16, rps18, rps19* (× 2)
	Large subunit ribosomal proteins	*rpl2* (× 2)*, rpl14, rpl16, rpl20, rpl22, rpl32, rpl33, rpl36*
	RNA polymerase subunits	*rpoA, rpoB, rpoC1, rpoC2*
	Transfer RNA	*trnA-UGC* (× 2)*, trnC-GCA, trnD-GUC, trnE-UUC, trnF-GAA, trnfM-CAU, trnG-GCC, trnG-UCC, trnH-GUG, trnI-CAU* (× 2)*, trnI-GAU* (× 2)*, trnK-UUU, trnL-CAA* (× 2)*, trnL-UAA, trnL-UAG, trnM-CAU, trnN-GUU* (× 2)*, trnP-UGG, trnQ-UUG, trnR-ACG* (× 2)*, trnR-UCU, trnS-GCU, trnS-GGA, trnS-UGA, trnT-GGU, trnT-UGU, trnV-GAC* (× 2)*, trnV-UAC, trnW-CCA, trnY-GUA*
Other genes	Conserved hypothetical chloroplast ORF	*ycf1* (× 2)*, ycf2* (× 2)*, ycf3*^a^*, ycf4*^a^
	Other proteins	*accD, ccsA, cemA, clpP, infA, matK*
Pseudogenes		*rpl23* (× 2)

^a^Genes associated with Photosystem I.

**Figure 2:**
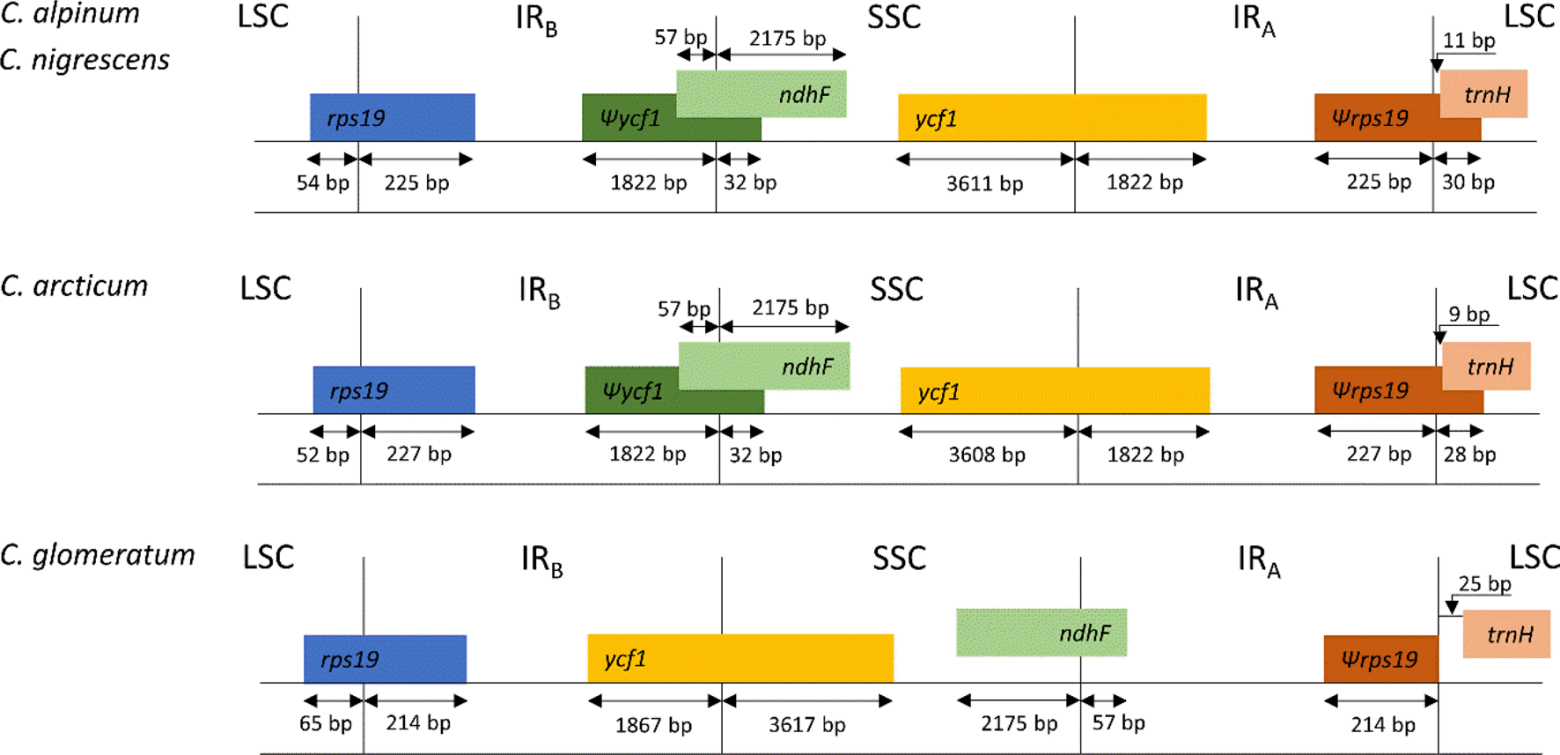
Comparison of LSC, SSC, and IR boundaries of four *Cerastium* chloroplast genomes.

The boundaries between IR and LSC/SSC regions were identified (Fig. 2). In the case of plastomes of *C. alpinum, C. arcticum,* and *C. nigrescens* the complete sequence of *ycf1* gene was located on the boundary between IR_A_ and SSC, and its incomplete copy on IR_B_/SSC boundary where it functions as a pseudogene (*Ψycf1*). *Ψycf1* was overlapped (89 bp) with the *ndhF* gene. The IR_A_/SSC boundary was located within *ycf1* sequence 1822 bp from its 5’ end. The IR_B_/LSC boundary was found within the *rps19* gene (52–54 bp from its 3’ end, depending on the species). Its shorter copy was located at the IR_A_/LSC boundary, where it acts as a pseudogene (*Ψrps19*). *Ψrps19* was overlapped (19 bp) with *trnH* gene. The *trnH* gene was near the IR_A_/LSC border (11 bp apart in case of *C. alpinum* and *C. nigrescens* and 9 bp for *C. arcticum*). The localization of IR and LSC/SSC boundaries was also analyzed for *C. glomeratum*. In the case of this species the analyzed boundaries were identified within the same genetic elements. The IR_B_/LSC boundary was located within the *rps19* gene (65 bp from its 3’ end) and pseudogene *Ψrps19* was found at the IR_A_/LSC boundary. However, *Ψrps19* did not overlap with *trnH*. Analysis of the IR_A_/SSC and IR_B_/SSC boundaries revealed the inversion of the entire SSC region in *C. glomeratum* cp genome. The IR_B_/SSC border was located within *ycf1* gene (1867 bp from its 5’ end) whereas the IR_A_/SSC was within the *ndhF* gene (57 bp from its 3’ end). The sequence for *Ψycf1* was not annotated in the analyzed plastome. Finally, the *trnH* gene was located 25 bp apart from the IR_A_/LSC border.

### Repetitive sequences and SSRs

**Figure 3:**
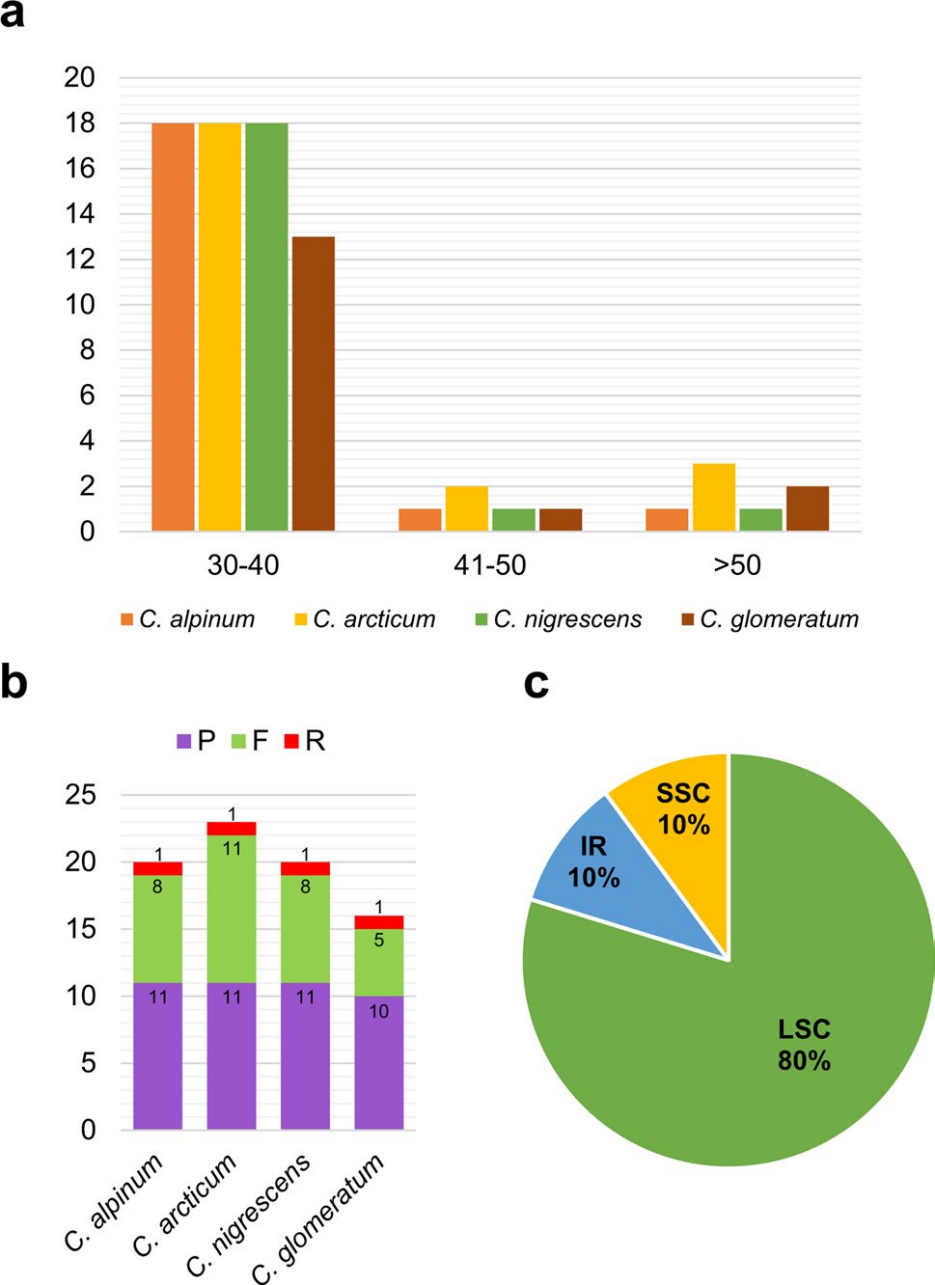
Number of repeat types and their distribution in four *Cerastium* species. (**a**) Length of the repeats; (**b**) types of repeats; (**c**) location of repeat sequences. F, P, R represent forward, palindromic and reverse repeats.

The analysis of genomic repeats in the cp genomes of studied *Cerastium* species (*C. alpinum*, *C. arcticum*, *C. nigrescens,* and *C. glomeratum* revealed 79 repetitive sequences with lengths ranging from 30 to 170 bp (Supplementary Table S2A–D). The number of repeats was the highest (23) in *C. arcticum* and the lowest (16) in *C. glomeratum*. Palindromic repeats dominated among identified sequences (from 47.8% in *C. arcticum* to 62.5% in *C. glomeratum*), followed by forward repeats (from 31.3% in *C. glomeratum* to 47.8% in *C. arcticum*) and reverse repeats (from 4.3% in *C. arcticum* to 6.3% in *C. glomeratum*) (Fig. 3b). No complementary repeats were found in analyzed chloroplast genomes. Most repeat sequences (80%) were found in the LSC region, and the remaining repeats were equally distributed (10%) in IR and SSC regions (Fig. 3c). Repeats with a length of 30–40 bp were the most frequent in each species (from 13 in *C. glomeratum* to 18 in *C. alpinum*, *C. arcticum,* and *C. nigrescens*) (Fig. 3a).

**Figure 4:**
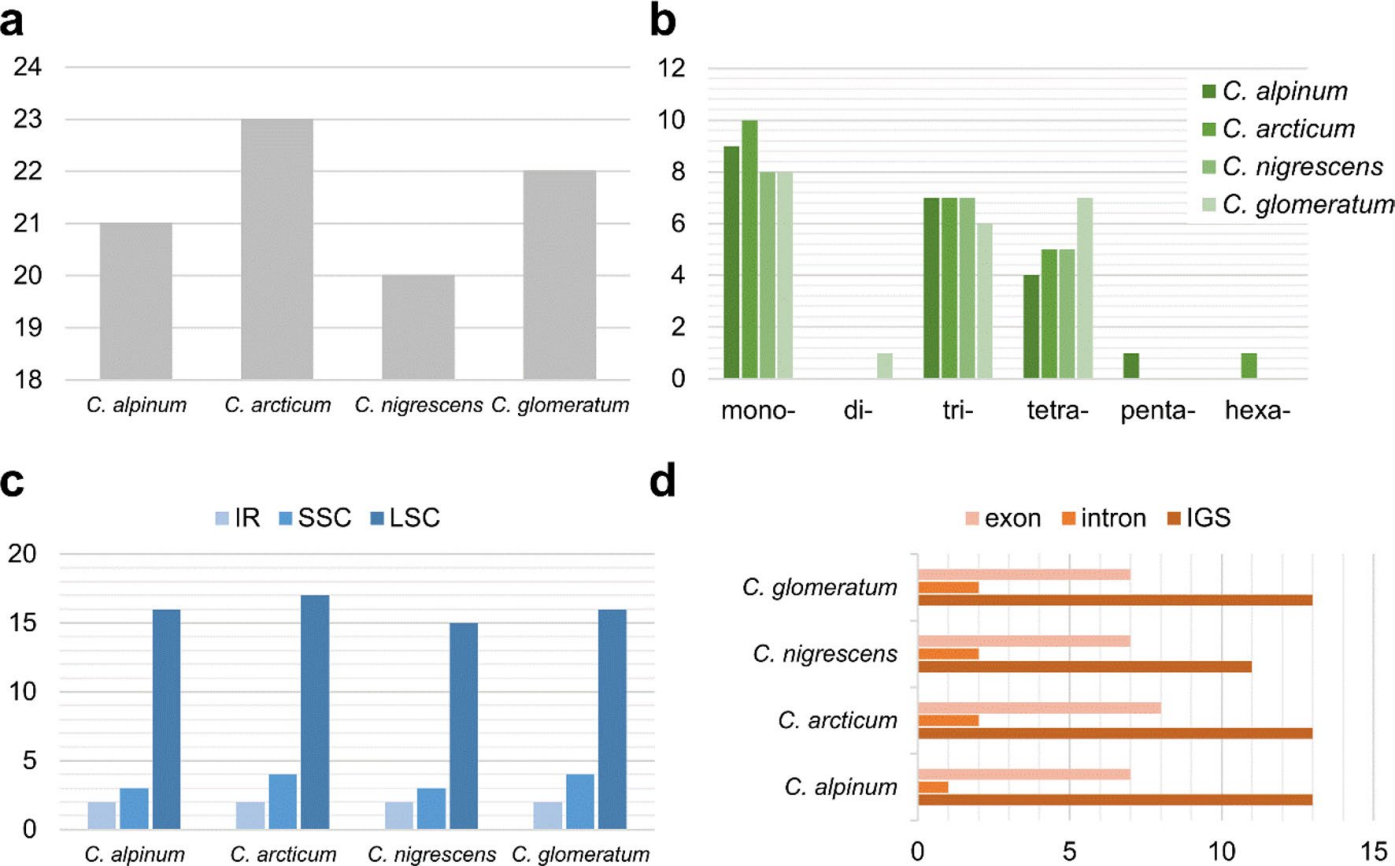
The distribution and type of simple sequence repeats (SSRs) in cp genomes of four *Cerastium* species. (**a**) Number of different SSRs types; (**b**) distribution of SSR motifs in different repeat class types; (**c**) location of different SSRs in IR, SSC and LSC regions; (**d**) partition of SSRs among IGS, introns and exons.

Application of the Phobos software revealed from 20 (*C. nigrescens*) to 23 (*C. arcticum*) chloroplast microsatellites (Fig. 4a), including mono-, di-, tri-, tetra-, penta- and hexanucleotide SSRs (Fig. 4b, Supplementary Table S3A–D). The mononucleotide SSRs, all composed of A/T repeat units, were the most common in each species with a frequency ranging from 36.4% (*C. glomeratum*) to 43.5% (*C. arcticum*). The second most common motif among identified SSRs was AAT/TAA with a frequency ranging from 27.3% (*C. glomeratum*) to 35% (*C. nigrescens*). Tetranucleotide SSRs, with frequency ranging from 19% (*C. alpinum*) to 31.8% (*C. glomeratum*) were composed of AAAT/TAAA, AATT/TTAA, ACCT/TCCA, AGAT/TAGA, and AAAG/GAAA motifs. Among identified chloroplast microsatellites there was only one SSR that contained a dinucleotide motif (AT/TA, *C. glomeratum*), one SSR with pentanucleotide motif (AATAT/TATAA, *C. alpinum*), and one SSR built of hexanucleotide motif (AAATCC/CCTAAA, *C. arcticum*). A substantial number of SSRs were identified in the LSC region (from 72.7% in *C. glomeratum* to 76.2% in *C. alpinum*), followed by SSC (from 14.3% in *C. alpinum* to 18.2% in *C. glomeratum*) and IR regions (from 8.7% in *C. arcticum* to 10% in *C. nigrescens*) (Fig. 4c). SSRs were mainly located within intergenic spacers (from 55% in *C. nigrescens* to 61.9% in *C. alpinum*), whereas the remaining microsatellites were distributed within exons (from 31.8% in *C. glomeratum* to 35% in *C. nigrescens*) and introns (from 4.8% in *C. alpinum* to 10% in *C. nigrescens*) (Fig. 4d).

### Synonymous (Ks) and non-synonymous (Ka) substitution rate analysis

**Figure 5:**
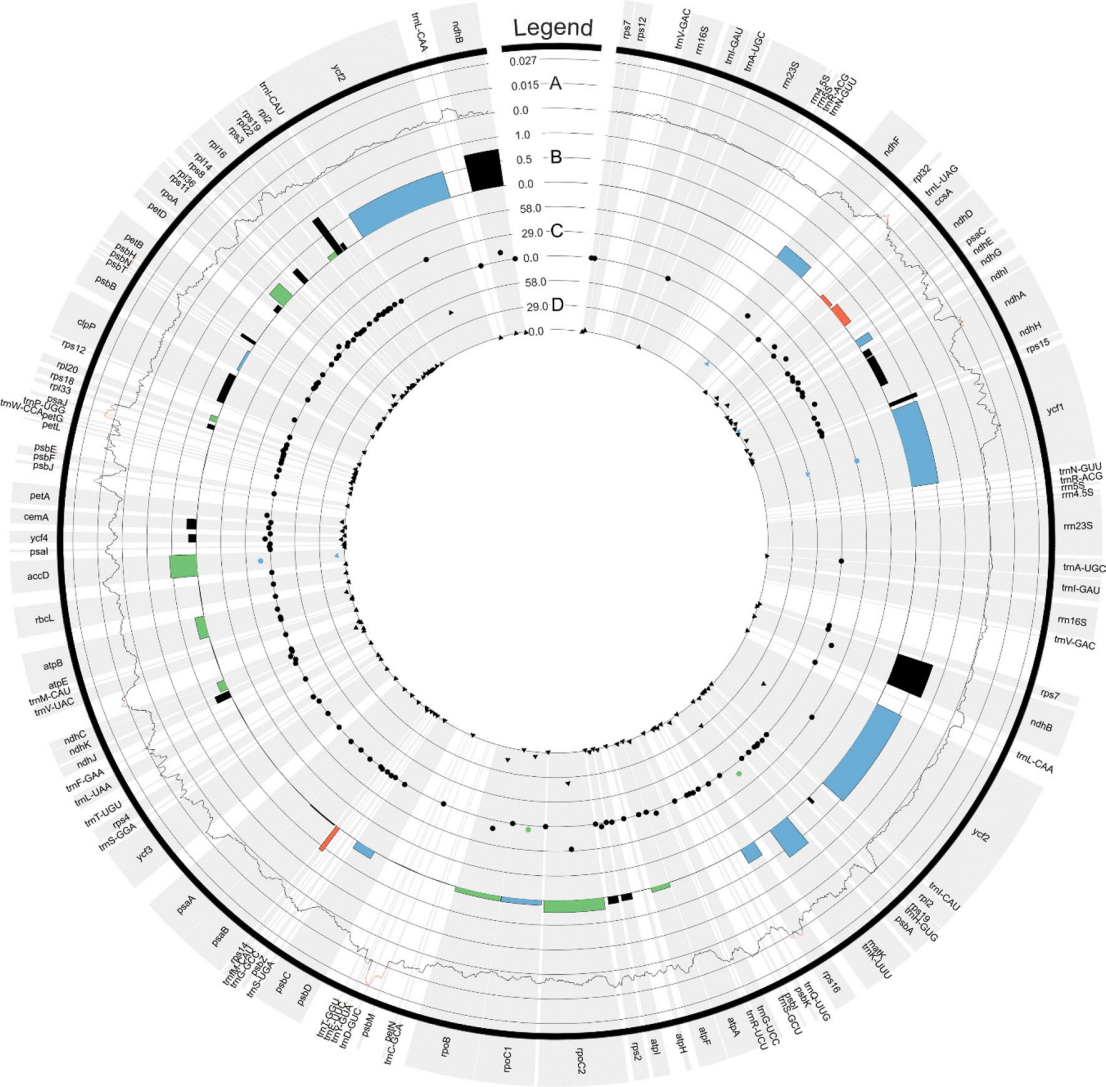
Circular visualization of the plastome comprehensive analyses of three *Cerastium* species (*C. nigrescens, C. arcticum,* and *C. alpinum*). The first outer track represents the chloroplast gene symbols. The second line track (**A**) shows haplotype diversity (π) values calculated for sliding window equal to 800 bp. The red part of the line plot depicts regions with the highest diversity (π > 0.015). Histograms (**B**) show comparative Ka/Ks ratio values for *Cerastium* species, where blue, red, green and black colors depict the dominant Ka/Ks values in *C. arcticum, C. nigrescens*, equal for *C. alpinum* and *C. nigrescens*, and equal for all three species, respectively. Both scatter plots show the number of potential C > U and U > C editing sites within each plastid gene (**C**,**D**, respectively). The colors describe higher numbers of RNA editing sites in *C. arcticum* (blue points) and *C. nigrescens* and *C. alpinum* (green points) in comparison to other compared species.

The substitution rate varied across genes in each functional group and ranged from 0 to 0.151 and from 0 to 0.0858 for Ka and Ks, respectively (Supplementary Table S4). The highest average value of Ka (0.0062) was noted in the group of “other genes” and the lowest (0.0012 and 0.0014) in genes related to the cytochrome b/f complex and photosystem II, respectively. The highest average value of Ks (0.0247) was noted in gene for RubisCO large subunit, and the lowest in genes associated with the small subunit of ribosome (0.0159) and subunits of ATP synthase (0.0160). In summary, no differences (Ka = 0 and Ks = 0) were observed in the sequences of 11 genes, whereas only synonymous substitutions (Ka = 0) were observed in 18 genes. The Ka/Ks ratio was less than 1 in all genes, excluding *ndhB* (2.7250 for *C*. *arvense*). Relatively high values of Ka/Ks were observed in *rpl22* (0.8673) for all studied species and in *rps14* (0.8776) for *C*. *arvense*. In the remaining cases, the values did not exceed 0.75 (Fig. 5).

### Genomic comparative and nucleotide diversity analyses

The MAUVE results revealed a highly conservative structure of chloroplast genomes of *C. alpinum*, *C. arcticum*, *C. nigrescens,* and *C. arvense* for which no rearrangements (inversions or translocations) were detected. Only in the case of *C. glomeratum* the opposite orientation of the whole SSC region was observed (Supplementary Fig. S2).

Nucleotide diversity (π) in the analyzed cp genomes of *Cerastium* species was determined at 0.00493. The results of sliding window analysis showed that the π value for studied *Cerastium* cp genomes varied from 0 to 0.02708 (Fig. 5). Nine highly variable (π > 0.015) regions were identified in analyzed cp genomes: *rpl32*–*trnL*-UAG, *ndhA* (intron), *rps16* (intron), *trnD-*GUC*–trnY-*GUA, *trnF*-GAA–*ndhJ*, *ndhC–trnV-*UAC, *petA–psbJ*, *psbE–petL,* and *trnP-*UGG*–psaJ*. The highest π value (0.02708) was observed for *trnD-*GUC*–trnY-*GUA region. All of these divergent hotspots were identified in non-coding regions i.e. intergenic spacers and introns. Furthermore, the majority of highly variable regions (7) were identified in LSC, followed by two such regions in SSC, and none in the IR region (Fig. 5).

### Prediction of RNA editing sites

Prediction of RNA editing sites with the use of PREPACT 3.0 tool revealed from 578 to 588 editing sites in 63 protein-coding genes (Fig. 5, Supplementary Table S5A–D). The lowest number of predicted RNA editing sites (578) was found for *C. alpinum* and *C. nigrescens*, whereas the highest was for *C. glomeratum*. In the case of the *C. arcticum* the number of RNA editing sites was 583. Among identified editing events both C to U and U to C conversions were found. In the case of 14 genes no such changes were identified. The C to U conversion accounted for 43.05% to 43.54% of total RNA editing sites, while U to C substitutions were responsible for 56.46% to 56.95% of the identified editing events. All predicted RNA editing sites resulted in non-synonymous mutations. Forty-seven (47.17–47.28%) percent of the substitutions were found at the first position of the codon, 53% (52.72–52.83%) were found at the second position, and none were found at the third position. Among predicted RNA editing events there were also conversions that involved two sites of RNA editing within one codon. Eighteen such editing events were identified in the case of *C. alpinum* and *C. nigrescens,* and 20 for *C. arcticum* and *C. glomeratum*. Most of these events involved conversions of UCU and UCC codons for serine (S) into CUU and CUC triplets for leucine (L) and back from leucine to serine, and also conversion of UUU and UUC for phenylalanine (F) to CCU and CCC for proline (P), and in the opposite direction i.e., from proline to phenylalanine. The highest number of predicted RNA editing sites were reported for *ycf1* (85–88), *ycf2* (77), and *rpoC2* (64–65) genes. The most often substitution in each species was phenylalanine (F) to leucine (L) change (16.48–16.75%), whereas P (proline) to F (phenylalanine) and R (arginine) to W (tryptophan) changes were observed with the lowest frequencies (0.353–0.358% and 0.881–0.896%, respectively). Additionally, the conversion of the termination codon UAA to CAA triplet encoding glutamine was found to be created by RNA editing in *ndhI* gene for *C. arcticum*.

Additionally, we conducted the same investigation for chloroplast genes of *C. arvense*. Unfortunately, due to incomplete sequences available for *rpl20*, *rpoB*, *rpoC1*, *rpoC2*, *ycf1,* and *ycf2,* these genes were not included in the analysis. In 17 out of 71 analyzed genes, we did not identify potential RNA editing sites. In the remaining 54 genes we found 286 editing sites (Fig. 5, Supplementary Table S5E). Further, for this species, both C to U and U to C conversions were found, but U to C edition dominated (56.46%). The highest number of substitutions were observed for the first (53.06%) and the second (46.94%) position of the codon, whereas they were absent in the third position. Analogous to the situation described above for *C. alpinum, C. arcticum, C. nigrescens,* and *C. glomeratum* also here, for C*. arvense,* among predicted RNA editing events we found conversions that involved two sites of RNA editing within one codon. There were seven situations in which CUU and CUA codons for leucine (L) were changed into UCU and UCA for serine (S), and backward from serine to leucine. The highest number of predicted RNA editing sites were identified within sequences for *matK* (40) and *ndhF* (37) genes. All the identified RNA edition events caused non-synonymous mutations. The change from phenylalanine (F) to leucine (L) was the most abundant substitution (18.53%), whereas leucine (L) to proline (P) and arginine (R) to cysteine (C) were observed with the lowest frequency (0.7%).

### Phylogenetic analysis

**Figure 6:**
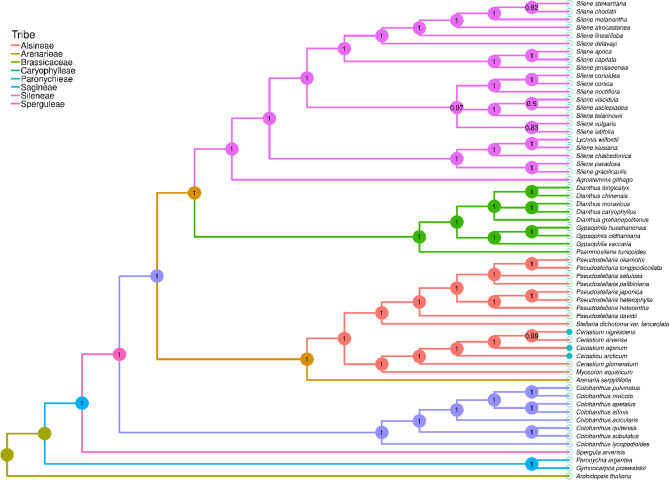
Phylogenetic tree (cladogram) based on sequences of sheared 71 protein-coding genes from five *Cerastium* species and 54 other Caryophyllaceae representatives using Bayesian posterior probabilities (PP). Bayesian PP are given at each node.

In the BI tree, a very high Bayesian posterior probability value (≥ 0.92) was reached in 96.4% of the nodes (53 out of 55). The reconstructed tree supported the taxonomic position of the studied species and revealed the following relationships: all *Silene* species together with *Lychnis wilfordii* formed one clad which gathered only representatives of Sileneae tribe; a second clad was formed by the representatives of the Caryophylleae tribe i.e., five *Dianthus* species, three representatives of genus *Gysophila* and *Psammosilene tunicoides*; a third clad consisted of all representatives of Alsineae tribe (eight *Pseudostellaria* species, *Stellaria dichotoma var. lanceolata*, *Myosoton aquaticum* and all studied *Cerastium* species which formed one subgroup; a fourth clad consisted of eight *Colobanthus* species (Sagineae tribe) whereas *Spergula arvensis* (Sperguleae) and *Paronychia argentea* with *Gymnocarpos przewalski* (Paronychieae) form two separate branches. The most diverged position on the tree was occupied by *A. thaliana* which was used here as an outgroup (Fig. 6).

**Figure 7:**
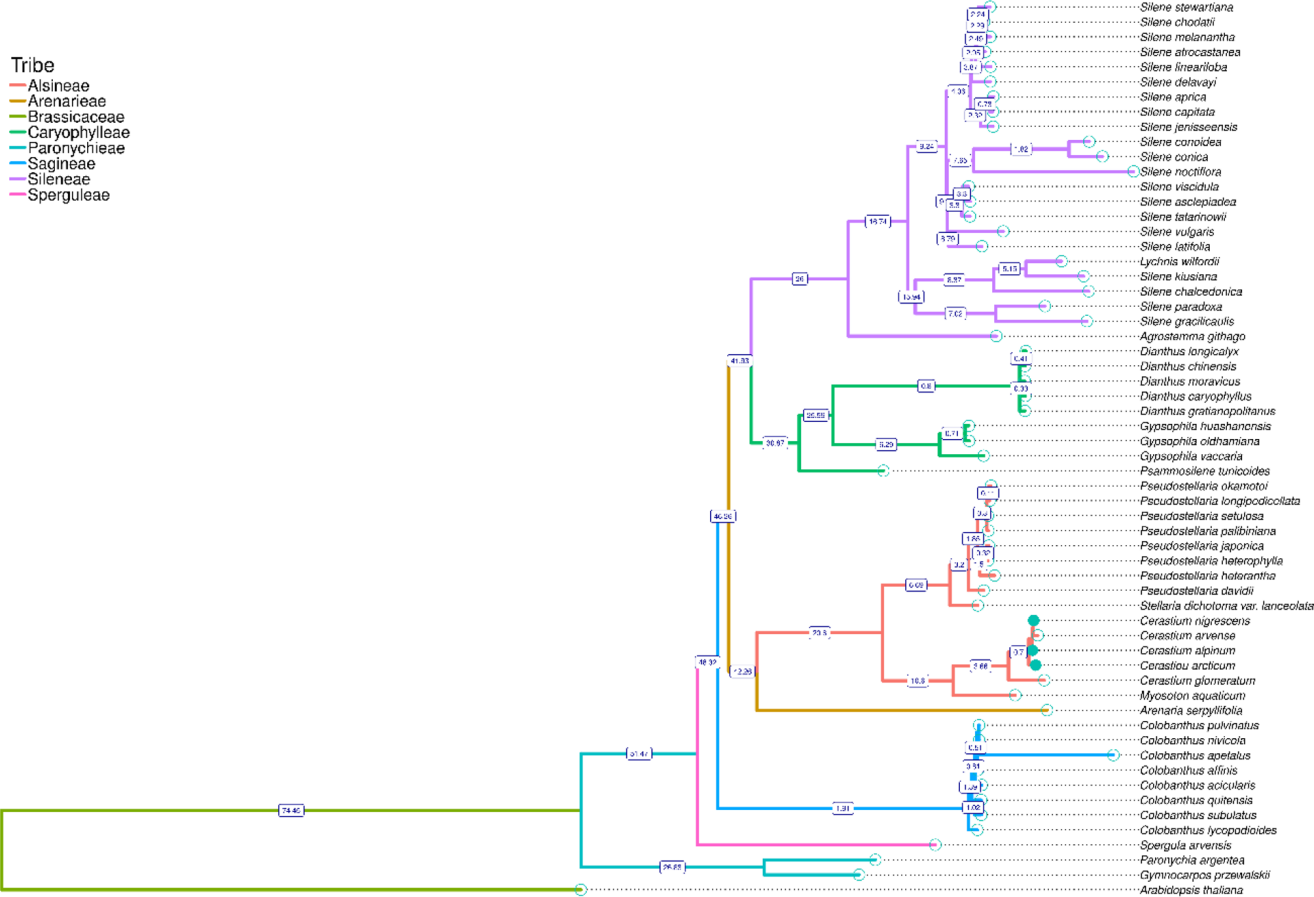
Divergence time estimation of selected Caryophyllaceae taxa. The numbers next to the nodes represent the divergence time (*Mya* millions years ago).

Results of divergence time estimation suggested that the family Caryophyllaceae started to diversify ca. 74.46 millions-years ago (Mya). Later, subsequent radiation within the family Caryophyllaceae occurred: ca. 51.47 Mya Sperguleae tribe splits from the other sister clades and at ca. 48.32 Mya diversification of Sagineae tribe was observed (represented here only by one genus *Colobanthus*). At ca. 46.26 Mya the evolutionary paths of Alsineae and Arenariae tribes diverged from Caryophylleae and Sileneae tribes. At ca 42.26 Mya Alsineae and Arenariae split apart and c.a. 41.93 Mya Caryophylleae split from Sileneae. Diversification events at the lower taxonomic level e.g. within tribe Caryophylleae, Sileneae and Alsineae started at 30.87, 26.0 and 20.6 Mya, respectively. The genus *Cerastium* began to diversify at 3.66 Mya (Fig. 7).

## Discussion

Chloroplast genomes are a relevant resource for many genomic and biotechnological applications^31^. Its unique features, like lack of recombination and slower mutation rate in comparison to nuclear genomes, make the chloroplast genome a frequently used source of data in evolutionary biology^32^. Moreover, common use of chloroplast genome in phylogeographic studies is observed due to its uniparental inheritance that exhibits geographical structure^33^.

Although the genus *Cerastium* consists of more than 200 species^1^, the availability of the genomic data for this group of plants is very limited and, to date there is only one complete chloroplast genome sequence in the NCBI database for *C. glomeratum*. There is also a chloroplast genome sequence for another *Cerastium* species (*C. arvense*), but due to the several gaps in the intergenic spacers and lack of complete sequences for six protein-coding genes (*rpl20*, *rpoB*, *rpoC1*, *rpoC2*, *ycf1,* and *ycf2*), create constraints for the utilization of this sequence. To fill the gap in the knowledge concerning the genomics of the genus *Cerastium* we sequenced and annotated the plastid genomes of three species: *C. alpinum*, *C. arcticum,* and *C. nigrescens*. The size of reported cp genomes ranged from 147,940 (*C. nigrescens*) to 148,722 bp (*C. arcticum*) and was similar to the plastome of *C. glomeratum* (148,643 bp) and other angiosperms^34^. All three studied cp genomes share the same gene content and order and typical quadripartite structure, with a pair of inverted repeats (IR) separated by a SSC and a LSC region). Length variation in cp genomes in different groups of plants is often caused by expansions and contractions of IR regions^35^. In extreme cases, IR regions were completely lost by chloroplast genomes of some algae^36^ or one of its copies is not observed in some representatives of leguminous plants^37^. Consequently, the analysis of the distribution of IR/LSC and IR/SSC borders became a standard element of plastome characteristics. Obtained results revealed that their locations may differ among various species, even between closely related genera^38^. Analysis of reported here chloroplast genomes of *C. alpinum, C. arcticum,* and *C. nigrescens* revealed that IR/LSC and IR/SSC boundaries were located within sequences of *ycf1* and *rps19* genes (Fig. 2), which is analogous to the situation observed for most angiosperms^39^. The location of IR boundaries was identical for *C. alpinum* and *C. nigrescens*, whereas a minor shift (two bases shift within *rps19* and three bases within the *ycf1* gene) was observed for *C. arcticum*. The length of IR and SSC regions in reported plastomes was very similar and ranged from 25,507 to 25,513 bp and from 16,850 to 16,861 bp, respectively. Higher variation was found for the LSC region where the difference between the longest and the shortest LSC is 782 bp (*C. arcticum* vs. *C. nigrescens*). Nevertheless, the sizes of all three plastome regions values are consistent with previous reports for other dicotyledons^40,41^. For comparative purposes, the IR borders within the chloroplast genome of *C. glomeratum* were also examined. In this case, more differences were observed. Although the IR borders were also located within the *rps19* and *ycf1* genes, the eleven base shift for *rps19* and 45 base shift for *ycf1* was found. Additionally, only one copy of *ycf1* can be found within *C. glomeratum* plastome at the IR_B_/SSC border as its incomplete copy (*ψycf1*) between IR_A_/SSC was not annotated. However, the main difference is associated with the opposite orientation of the whole SSC region. This interesting phenomenon was originally reported for *Phaseolus* vulgaris^42^. The author with the use of restriction enzyme analysis revealed, that the individual plants' chloroplast DNA demonstrates a type of heteroplasmy in which the plastomes occurs in two equimolar states (i.e., inversion isomers) that differ in the orientation of the SSC region. Later this phenomenon was confirmed in other species, e.g., *Heterosigma akashiwo*^43^, *Lasthenia burkei*^44^, and *Artemisia frigida*^45^.

Chloroplast genomes of *C. alpinum*, *C. arcticum,* and *C. nigrescens* contained an identical set of 113 genes which appeared to be identical with *C. arvense*. In the case of the cp genome of *C. glomeratum* lack of the *psbL* gene was noticed during the analyses, but reannotation of the plastome allowed us to identify the *psbL* sequence between *psbJ* and *psbF* genes. Furthermore, two additional genes, i.e., *infA* (coding translation initiation factor I) and *rpl23* (encoding ribosomal protein L23) were not annotated in *C. glomeratum* plastome. Detailed analysis of the chloroplast genome for the species enable identification of these sequences, but their pseudogenization (i.e., the presence of internal, premature termination codons) was the most probable reason why their annotations were not considered by the original authors of the sequence. In the case of *C. alpinum*, *C. arcticum,* and *C. nigrescens rpl23* was also identified as a pseudogene, whereas a complete sequence of *infA* gene was found and annotated. Loss of the *infA* gene was also observed in other species within the Caryophyllales^46^. In some cases, the *infA* gene was found to be a pseudogene, i.a. in *Nicotiana tabacum*^47^, *Arabidopsis thaliana*^48^, *Oenothera elata*^41^ and several *Allium* species^49^. In the chloroplast genomes of another Caryophyllaceae representative, i.e. *Dianthus superbus var. longicalyncinus*, both *infA* and *rpl23* were retained as pseudogenes^50^. Pseudogenization of the *rpl23* gene was also previously reported in various species, i.a. within the genus *Triticum*^51^, *Hordeum*^52^ and *Secale*^53^. The studied *Cerastium* cp genomes had a GC-content of 36.46–36.52%, which is comparable with other Caryophyllaceae – 36.32% in *Dianthus caryophyllus*^54^, 36.4% in *Silene jenisseensis*^55^, 36.5% in *Pseudostellaria palibiniana*^56^, *P. okamotoi*^57^, *P. heterophylla*^58^, *P. longipedicellata*^59^ and *Gymnocarpos przewalskii*^60^ and 36.7% in *Colobanthus quitensis*^61^.

The repeat regions of the genomes are of particular importance in sequence rearrangement and recombination^62^. The genomic repeats identified within chloroplast genomes of *C. alpinum*, *C. arcticum*, *C. nigrescens,* and *C. glomeratum* ranged from 30 to 170 bp in length and they were identified predominantly (56.3–69.6%) within non-coding regions. Similar values were reported in other Caryophyllaceae, such as *C. quitensis* (53.3%^63^;), *Silene capitata* (56.0%) and *Lychnis wilfordii* (69.2%)^64^. The majority of the repeats (78–90%) in all four *Cerastium* genomes are between 30 and 40 bp in length. Similar values were reported in other angiosperms—legumes (*Glycine*, *Lotus*, *Medicago*^65^) and cotton (*Gossypium hirsutum*^66^).

Chloroplast simple sequence repeats, or microsatellites, are repetitive genomic elements that typically consist of tandemly repeated multiple copies of mono- to hexanucleotide motifs which are usually found in the non-coding regions^67^. Due to their high abundance, random distribution within the genome and high polymorphism information content, they are also widely used for high-throughput genotyping^68^. These markers proved their usefulness in population genetics and evolutionary studies^69,70^. In the analyzed plastomes of four *Cerastium* species, the mononucleotide (A/T) repeats were the most abundant SSR motif (36.4–43.5%). The dominance of mononucleotide chloroplast SSRs has been also observed in other Caryophyllaceae, where it ranged from 44.8% in *Colobanthus apetalus*^63^ or 55.3% in *C. lycopodioides*^71^ up to 76.8% in *Silene capitata* or 77.6% in *Lychnis wilfordii*^64^. In turn, di- (AT/TA), penta- (AATAT/TATAA) and hexanucleotide (AAATCC/CCTAAA) microsatellites were least abundant, and only one such element was identified in *C. glomeratum*, *C. alpinum,* and *C. arcticum*, respectively.

The synonymous (Ks) and non-synonymous (Ka) substitution rate and their ratio (Ka/Ks) are important parameters in gene evolution studies^72^. Generally, in most of the coding regions synonymous nucleotide substitutions dominate over non-synonymous changes^73^. This was also observed in our study, where Ks values dominated over Ka which resulted in high sequence conservation. Nevertheless, there were also sequences for which considerable variation was found due to the high Ka values. The highest Ka values were observed for *rpl32* (average Ka = 0.0151) and *matK* (average Ka = 0.0134). High variation of the *matK* sequence has been widely documented and it is recognized as one of the most promising barcoding sites for systematic and evolutionary studies in plants^74,75^. There are also studies reporting high genetic diversity in the immediate vicinity of the *rpl32* gene (*ndhF–rpl32* or *rpl32–trnL*)^76,77^ and the role of *rpl32* gene in the evolution of chloroplast genomes which involve its complete loss, substitution or transfer to the nucleus (for review see^78^). Assessment of the ratio of nonsynonymous (Ka) to synonymous (Ks) substitution is widely accepted approach used to infer about the direction of the sequence evolution at the protein level (Ka/Ks > 1 indicates a positive selection, Ka/Ks < 1 indicates a negative or purifying selection, whereas Ka/Ks = 1 indicates a neutral evolution)^79,80^. Protein functions are maintained through purifying selection, whereas positive selection favors new gene variants which may be beneficial for organism adapting to changing environmental conditions. In the case of our study, Ka/Ks ratio of all genes was less than 1, except for *ndhB* (2.7250 for *C. arvense*), implying that this gene evolved at a faster rate and underwent positive selection. The same pattern of selection (Ka/Ks > 1.0) for *ndhB* gene was also reported in various species representing the family Gentianaceae (*Gentiana lawrencei*^81^), Orchidaceae (*Calanthe delavayi*^82^) and Cupressaceae (*Cupressus* and *Juniperus* species^83^). The group of *ndh* genes, encoding subunits of NADH dehydrogenase, play a key role in the use of light energy and electron transfer chain to produce ATP, an essential component for photosynthesis^84^. Chloroplast NADH dehydrogenase is sensitive to strong light stress and can protect plants from photoinhibition or photooxidation stress by stabilizing the NADH complex and preventing drought-related declines in photosynthetic rate and growth delay^85^. These observations may suggest that NADH dehydrogenase genes are involved in adaptation to environmental stresses by optimization of photosynthesis. An excess of functionally adaptive amino acid substitutions within NADH dehydrogenase genes was described previously for Poaceae^86^. Authors observed there the signals of positive selection acting on one-third of all chloroplast protein-coding genes (25 out of 76), including nine of the eleven genes encoding subunits of NADH dehydrogenase. In the case of our study, the signal of positive selection detected for the *ndhB* gene in *C. arvense* which might be interpreted as one of the mechanisms of physical adaptation which enabled this cosmopolitan species to colonize vast areas of Europe and North America.

Highly variable sequences found within chloroplast genomes appeared as a common source of molecular markers suitable for phylogenetic analyses and species identification^87^. Although traditional barcoding chloroplast regions, like *matK, rbcL* or intergenic spacer *trnH-psbA* revealed lower than expected genetic diversity, our genome-wide comparative analysis of plastomes of four *Cerastium* species (*C. alpinum, C. arcticum, C. nigrescens,* and *C. glomeratum*) allowed us to identify nine fast evolving regions. Among these divergent hotspots (π > 0.015) there were seven regions (*trnD-*GUC*–trnY-*GUA, *trnF*-GAA–*ndhJ*, *ndhC–trnV-*UAC, *petA–psbJ*, *psbE–petL*, *trnP-*UGG*–psaJ* and intron within *rps16* sequence) located within LSC region and two others (*rpl32*–*trnL*-UAG and intron within *ndhA* sequence) identified within SSC region. To the best of our knowledge, none of these chloroplast genome regions have been used to date for phylogeny reconstruction within the genus *Cerastium*. Nevertheless, there are several phylogenetic studies performed within various groups of plant species, including the family Caryophyllaceae, in which at least some of the regions listed above were used, e.g. intron of *rps16*^88^, *petA–psbJ*^89^ or *rpl32*–*trnL*-UAG^90^.

RNA editing is one of the most important post-transcriptional modifications which mainly occurs in mitochondrial and chloroplast transcripts^91,92^. RNA editing is described as a process involved in the correction of a missense mutation of genes at the RNA level. This mechanism could alter the nucleotide sequence through insertion, deletion, or substitution of nucleotides^93,94^ to preserve the function of encoded proteins^95^. The first report of RNA editing was documented for the *cox2* gene in the protozoan parasite *Trypanosoma brucei*^96^, whereas in plants RNA editing was first discovered in the sequence of *cox2* of *Triticum aestivum*^97^ and then in *rpl2* in maize^98^. Several editing sites have been reported in many other species, i.a. *A. thaliana*^93^, *N. tabacum*^99^, *Oryza sativa*^100^, *Pisum sativum*^101^ and *Manihot esculenta*^102^. RNA editing that converts cytidine into uridine (C into U) is widespread in plant organelles and occurs mostly at the first or second positions of codons^103^. Whereas the reverse U to C conversions is more restricted in occurrence. In studied *Cerastium* species the presence of both C to U and U to C editing has been revealed. RNA editing by U to C is rather rare in terrestrial plants, but it has been found in some species i.a. *A. thaliana*^104^, hornworts^105^, lycophytes^106^ and ferns^107^.

One of the plant groups that has been intensively studied in terms of its phylogeny is the family Caryophyllaceae. Traditionally, Caryophyllaceae was divided into three subfamilies: Alsinoideae, Caryophylloideae, and Paronychioideae^108^. However, the traditional taxonomy of the family encountered many difficulties, i.e., most of the genera appeared to be polyphyletic probably because many of the morphological characters evolved in parallel^109^. More recently, a new classification of Caryophyllaceae family based on three chloroplast regions (*matK, trnL-trnF,* and *rps16*) was proposed which divided this group into 11 tribes^110^. Unfortunately, only two *Cerastium* species (*C. arvense* and *C. fontanum*) were represented in this study and based on their molecular characteristics they were nested within the Alsineae tribe, together with representatives of the following genera: *Stellaria, Pseudostellaria, Myosoton, Plettkea, Holosteum, Moenchia,* and *Lepyrodictis*. *Cerastium* is one of the Caryophyllaceae genera whose structure is still intensively debated. Even determining the number of species distinguished within this group of plants is problematic and vary from 60^111^ or 100^3,112^ up to 200^113^ species. Phylogenetic analyses employing multiple nuclear and plastid DNA sequences have established *Cerastium's* monophyly^13,114^. However, there are still some issues associated with *Cerastium* systematics that need clarification, for example, the status of the *C. alpinum*–*C. arcticum* complex which includes *C. alpinum, C. arcticum,* and *C. nigrescens*. Several evolutionary lineages were identified within that complex in earlier research based on morphology, isozymes, and DNA markers^6,10,19^. It was reported that the origin and evolution of these taxa are most likely related to the fluctuations of ice sheet range during the Quaternary glaciations which caused the extensive migrations of the species and enabled multiple hybridization and introgression events^11,19,115^. This hypothesis is consistent with the results of studies reporting no variation in chloroplast *trnL-trnF* and *psbA-trnH* sequences among representatives of the arctic-alpine *C. alpinum*–*C. arcticum* complex and members of the boreal-temperate *C. tomentosum* and *C. arvense* groups^13^.

In our study, phylogenetic analysis was based on 71 concatenated protein-coding gene sequences. Revealed phylogenetic relationships between analyzed representatives of the Caryophyllaceae family were in concordance with the taxonomic position of studied species and previous phylogenies of this group^109,116^. Moreover, obtained results allowed us to undoubtedly discriminate all analyzed species, including five representatives of the genus *Cerastium* (*C. alpinum, C. arcticum, C. nigrescens, C. glomeratum,* and *C. arvense*). This is in agreement with the previous observation that a phylogenetic network that combines several genes is preferable to a single-gene tree, as the latter is typically insufficient to reveal reliable phylogenetic relationships^117^. All *Cerastium* species were gathered in one clade, but *C. glomeratum* appeared to be the most divergent from the other species.

Our divergence time analysis confirmed the results of the previous studies on molecular and temporal diversification of the Caryophyllace family. Analogous to the results of the latest research based on nuclear ITS region and four plastid sequences (*matK*, *rbcL*, *rps16* and *trnL*-F)^118^ our studies suggested that the family Caryophyllaceae began to diversify before the end of Crecateous (ca. 74.46 Mya) and this process continued through the Paleogene and Neogene with the highest intensity of the diversification in the last 10 Mya^119^. According to our observations Alsineae tribe, which includes the genus *Cerastium*, started to diversify at 20.6 Mya, whereas the beginning of that process for the genus *Cerastium* was dated on ca. 3.66 Mya. Our results suggested that *C. glomeratum* split earliest from the other representatives of this genus, whereas the other species appeared to be on the early stages of diversification. The high similarity of studied *Cerastium* plastome sequences may be treated as possible evidence for weak barriers to breeding between these species which enabled spontaneous hybridization between them. Previously, interspecific hybridization events were reported for many *Cerastium* species^8,120^. Although a close relationship between *C. nigrescens* and *C. arcticum* was previously reported^10,18,19^, our study suggested a more divergent character of more geographically distant *C. arcticum* and closer genetic relationships between *C. nigrescens* and *C. alpinum*. These observations and results of previous studies pointing to possible hybridization between these two sympatric species (*C. nigrescens* and *C. alpinum*)^11^ showed the complexity of evolution which can take place across a broad range of scenarios and spatial circumstances^121^.

*C. arvense* was unexpectedly grouped with species from the *C. alpinum*–*C. arcticum* complex. This is probably because the publicly available partial sequence of *C. arvense* chloroplast genome that we used in phylogenetic studies lacked complete sequences of six genes (*rpl20*, *rpoB*, *rpoC1*, *rpoC2*, *ycf1,* and *ycf2*) thus the phylogeny reconstruction was performed on the limited number (71) of plastid genes. The absence of these genes might be then responsible for the underestimation of genetic divergence between *C. arvense* and other *Cerastium* species. The application of a complete sequence of chloroplast genome appeared here as the alternative method for distinguishing the true phylogenetic relationships between these closely related taxa. This approach has already proved its usefulness for taxa with a relatively short time since the divergence event or a low rate of evolution resulted in low sequence variation^31,122^. Nevertheless, in both cases resequencing of *C. arvense* is required.

Although complete chloroplast genomes of three *Cerastium* species (*C. alpinum, C. arcticum* and *C. nigrescens*) were reported and characterized here for the first time further research is required to investigate and finally resolve the taxonomic issues associated with the genus *Cerastium* and the *C. alpinum*–*C. arcticum* complex. Subsequent studies should include not only analyses of chloroplast genomes but also nucleic regions because when hybridization and polyploidy are common the resolution that chloroplast genome sequence can provide for phylogenomics research may be limited^123^. Nevertheless, our results proved the suitability of chloroplast genome sequences as reliable and effective DNA barcodes for *Cerastium* species.

## Conclusion

The chloroplast genomes of *Cerastium alpinum*, *C*. *arcticum,* and *C*. *nigrescens* were sequenced and characterized for the first time. The reported chloroplast genomes appeared to be highly conserved in terms of the gene content and order as well as their quadripartite structure. Highly divergent regions (*rpl32*–*trnL*-UAG, *ndhA* intron, *rps16* intron, *trnD-*GUC*–trnY-*GUA, *trnF*-GAA–*ndhJ*, *ndhC–trnV-*UAC, *petA–psbJ*, *psbE–petL* and *trnP-*UGG*–psaJ*) and microsatellite sequences that could be potentially used as markers in genetic diversity or phylogenetic studies were identified. Reconstruction of phylogenetic relationships within the family Caryophyllaceae confirmed the previously reported systematic relations within that group of plants and supported the position of *Cerastium* species as a separate clad within the tribe Alsineae. Although obtained data provide insight into the evolution and biogeographic history of the genus *Cerastium* further studies are needed to finally elucidate the relationships between species from the *C. alpinum*–*C. arcticum* complex.

## Methods

### Plant material, DNA extraction and chloroplast genome sequencing

The research material consisted of three *Cerastium* species–*C. alpinum*, *C. arcticum,* and *C. nigrescens*. Fresh leaves of *C. alpinum* and *C. arcticum* were harvested from plants grown from seeds in a greenhouse (Department of Plant Physiology, Genetics and Biotechnology, University of Warmia and Mazury in Olsztyn, Poland). The seeds of *C. alpinum* were collected in 2020 in Babia Góra National Park (Poland) after obtaining permission from the Polish Ministry of Environment. In the case of *C. arcticum*, seeds were collected by Michał Węgrzyn from the Institute of Botany of Jagiellonian University in Kraków, Poland, during the Arctic expedition to Nicolaus Copernicus University Polar Station in Spitsbergen in 2012. In turn, *C. nigrescens* individuals were collected by Keith W. Larson from Climate Impacts Research Centre, Umeå University, Sweden, in Nuolja massif (Sweden) and delivered to Olsztyn in dried form. The species identification included analysis of both vegetative and generative organs. In the case of *C. alpinum* and *C. arcticum* identification was performed by Irena Giełwanowska, whereas *C. nigrescens* status was verified by Keith W. Larson. Voucher specimens of each studied species have been deposited in the Vascular Plants Herbarium of the Department of Botany and Nature Protection at the University of Warmia and Mazury in Olsztyn, Poland (OLS), under the following numbers: *C. alpinum* (No. OLS 33837), *C. arcticum* (No. OLS 33840) and *C. nigrescens* (No. OLS 33841). The photographs of the representatives of each studied species were provided as the supplementary material: *C. alpinum* (Supplementary Fig. S3), *C. arcticum* (Supplementary Fig. S4) and *C. nigrescens* (Supplementary Fig. S5).

Total genomic DNA was extracted from the fresh or dried material of a single plant using Maxwell 16 LEV Plant DNA Kit (Promega, Madison, WI). The amount and purity of isolated DNA was estimated spectrophotometrically (NanoDrop ND-1000 UV/Vis; NanoDrop Technology). Additionally, the quality of DNA was verified on 1.5% (w/v) agarose in the presence of 0.5 µg/ml ethidium bromide (wavelength 300 nm; Ultra-Lum EB-20 Electronic UV Transilluminator).

The appropriate genome libraries (library kit: TruSeq DNA PCR Free (350), prepared from high-quality genomic DNA, were sequenced on Illumina NovaSeq6000 platform (Illumina Inc., San Diego, CA, USA) with a 150 bp paired-end read.

### Annotation and genome analysis

The quality of raw reads was checked with the FastQC tool. Raw reads were trimmed (5 bp of each read end, regions with more than 5% probability of error per base) and mapped to the reference chloroplast genome of *C. glomeratum* (NC_066897) using Geneious v.R7 software^124^ with medium–low sensitivity settings. The details on subsequent procedures for chloroplast genome assembly and annotation were described in our previous study^78^. The chloroplast genomes were annotated using PlasMapper^125^ with manual adjustment and circular maps of chloroplast genomes were drawn using the OrganellarGenome DRAW tool^126^. Each chloroplast genome assembly was validated using GetOrganelle v.1.7.7.0^127^.

Additionally, to check for the possible presence of heteroplasmy variant calling analysis was performed in Geneious software using “Find Variations/SNPs (Single Nucleotide Polymorphism)” feature with the following parameters: minimum variant frequency = 0.1; minimum coverage = 10, p-value cut off = 0.0001 and default values for the remaining parameters.

### Genomic repeats and SSR analysis

The genomic repeats, including forward, reverse, palindromic and complementary sequences were identified using REPuter software^128^ with the following settings: minimal repeat size of 30 bp, Hamming distance of 3, and 90% sequence identity. Chloroplast simple sequence repeats (SSR), also called microsatellites, were identified in Phobos v.3.3.12^129^. Only perfect SSRs with a motif size of one to six nucleotide units were considered. Additionally, we applied the standard thresholds for chloroplast SSRs’ identification^130^: minimum number of repeat units were set to 12, 6, 4, 3, 3, and 3 for mono-, di-, tri-, tetra-, penta- and hexanucleotides, respectively. A single IR region was used to eliminate the influence of doubled IR regions, and redundant results were deleted manually.

### Comparative analysis of chloroplast genomes

Chloroplast genome sequences of three *Cerastium* species (*C. alpinum, C. arcticum, C. nigrescens*) reported in this paper and plastome sequence of *C. glomeratum* (NC_066897) and *C. arvense* (MH627219) acquired from NCBI database were used for the genome synteny analysis which was performed with the use of MAUVE v.1.1.1^131^. Furthermore, the sequences were aligned in MAFFT v.7.310^132^ to perform sliding window analysis and evaluate nucleotide diversity (π) in chloroplast genomes using DnaSP v.6.10.04^133^. The step size was set to 50 base pairs, and the window length was set to 800 base pairs. Here, only complete chloroplast genome sequences were used—*C. arvense* plastome which has several gaps in its sequence was excluded from this analysis. The results were visualized with the CIRCOS software package v.0.69–9^134^.

The selective pressure for genes identified in chloroplast genomes of *C. alpinum, C. arcticum, C. nigrescens*, *C. glomeratum,* and *C. arvense* was also analyzed. A total number of 77 protein-coding genes were selected for which synonymous (Ks) and non-synonymous (Ka) substitution rates, as well as Ka/Ks ratio, were estimated using DnaSP v.6.10.04. *Cerastium glomeratum chloroplast* genome was used as a reference. During the analyses, lack of *psbL* gene was noticed in *C. glomeratum*. Reannotation of the *C. glomeratum* plastome allowed us to identify the sequence for this lacking gene in its traditional position i.e., between *psbJ* and *psbF* (detailed location: 61391..61507). In the *C. arvense* cp genome all genes which were annotated in plastomes of *C. alpinum, C. arcticum, C. nigrescens* were also present, but unknown nucleotides (n) were recorded in six (*rpl20*, *rpoB*, *rpoC1*, *rpoC2*, *ycf1,* and *ycf2*), therefore these sequences were excluded from calculations for this species. The results were visualized with the CIRCOS software package v.0.69-9^134^.

The junction sites between LSC, SSC, and IRs regions were also identified and compared. Additionally, data on the codon usage distribution was acquired from the Geneious v.R7 statistic panel.

### Prediction of RNA editing sites

Potential RNA editing sites in the protein-coding genes from chloroplast genomes of *C. alpinum, C. arcticum, C. nigrescens*, *C. glomeratum,* and *C. arvense* were predicted using PREPACT 3.0 tool^135^. *Arabidopsis thaliana* (NC_000932) was used as a reference for BLASTx prediction, both forward (C to U) and reverse (U to C) editing options were selected, while the remaining settings were kept at default (0.001 e-value cutoff and 30% filter threshold). In the case of *C. arvense rpl20*, *rpoB*, *rpoC1*, *rpoC2*, *ycf1,* and *ycf2* genes were excluded from the analysis as unknown nucleotides (n) were recorded in their sequences. The results were visualized with the CIRCOS software package v.0.69–9^134^.

### Phylogenetic analysis

Chloroplast genome sequences of three *Cerastium* species (*C. alpinum, C. arcticum,* and *C. nigrescens*) reported in this paper, as well as 56 plastomes of other representatives of family Caryophyllaceae (including *C. glomeratum* and *C. arvense*) and *A. thaliana* (outgroup), were used for phylogenetic analysis (Table 3). Initially, the sequences of 71 protein-coding genes shared by all these species were extracted using a custom R script. Then, the concatenated sequences of 71 genes were aligned in MAFFT v7.310 and used for phylogeny reconstruction by Bayesian Inference (BI). The Mega v.7 software^136^ was used to determine the best-fitting substitution model, and the GTR + G + I model was selected. The BI analysis was conducted using MrBayes v.3.2.6^137,138^, according to the parameter’s settings described in our previous paper^63^. The obtained phylogenetic tree was used as a starting tree for divergence time analysis performed using RelTimeML feature in MEGA 7 with GTR model. The divergence time between *Cerastium arvense* and *Myosoton aquaticum* (6.2–38.1 Mya), *Arenaria serpyllifolia* and *Pseudostellaria japonica* (20.3–83.4 Mya) and *Dianthus chinensis* and *Silene latifolia* (20.3–46.7 Mya) obtained in TimeTree^139^ were used as calibration constraints in calculations.

**Table 3:** GenBank accession numbers and references for chloroplast genomes used in this study. Species list arranged alphabetically.

Species	Accession number	Species	Accession number
*Arabidopsis thaliana*	NC_000932	*Pseudostellaria heterantha*	NC_058231
*Agrostemma githago*	NC_023357	*Pseudostellaria heterophylla*	NC_044183
*Arenaria serpyllifolia*	NC_065316	*Pseudostellaria japonica*	OP526393
*Cerastium alpinum*	QC696752	*Pseudostellaria longipedicellata*	NC_039454
*Cerastium arcticum*	QC696753	*Pseudostellaria okamotoi*	NC_039974
*Cerastium arvense*	MH627219	*Pseudostellaria palibiniana*	NC_041166
*Cerastium glomeratum*	NC_066897	*Pseudostellaria setulosa*	NC_041462
*Cerastium nigrescens*	QC696754	*Silene aprica*	MN097700
*Colobanthus acicularis*	NC_053724	*Silene asclepiadea*	NC_061183
*Colobanthus affinis*	NC_053722	*Silene atrocastanea*	NC_061184
*Colobanthus apetalus*	NC_036424	*Silene capitata*	NC_035226
*Colobanthus lycopodioides*	NC_053721	*Silene chalcedonica*	NC_023359
*Colobanthus nivicola*	NC_053720	*Silene chodatii*	NC_061185
*Colobanthus pulvinatus*	NC_053719	*Silene conica*	NC_016729
*Colobanthus quitensis*	NC_028080	*Silene conoidea*	NC_023358
*Colobanthus subulatus*	NC_053723	*Silene delavayi*	NC_061186
*Dianthus caryophyllus*	NC_039650	*Silene gracilicaulis*	NC_061187
*Dianthus chinensis*	NC_053731	*Silene jenisseensis*	MN723869
*Dianthus gratianopolitanus*	LN877392	*Silene kiusiana*	NC_048886
*Dianthus longicalyx*	NC_050834	*Silene latifolia*	NC_016730
*Dianthus moravicus*	LN877396	*Silene lineariloba*	NC_061188
*Gymnocarpos przewalskii*	NC_036812	*Silene melanantha*	NC_061189
*Gypsophila huashanensis*	OP094658	*Silene noctiflora*	NC_016728
*Gypsophila oldhamiana*	NC_058757	*Silene paradoxa*	NC_023360
*Gypsophila vaccaria*	NC_040936	*Silene stewartiana*	NC_061190
*Lychnis wilfordii*	NC_035225	*Silene tatarinowii*	NC_061191
*Myosoton aquaticum*	MZ570968	*Silene viscidula*	NC_061192
*Paronychia argentea*	NC_066008	*Silene vulgaris*	NC_016727
*Psammosilene tunicoides*	MK684403	*Spergula arvensis*	NC_041240
*Pseudostellaria davidii*	OP526392	*Stellaria dichotoma var. lanceolata*	MN718731

### Ethics declarations

Authors confirm that the use of plants in the present study complies with international, national and/or institutional guidelines.

### Supplementary Information

Supplementary Figure S1.

Supplementary Figure S2.

Supplementary Figure S3.

Supplementary Figure S4.

Supplementary Figure S5.

Supplementary Legends.

Supplementary Table S1.

Supplementary Table S2.

Supplementary Table S3.

Supplementary Table S4.

Supplementary Table S5.

## Data Availability

The complete chloroplast genomes reported in this paper have been submitted to the NCBI database under the following accession numbers: QC696752 for *C. alpinum*, QC696753 for *C. arcticum,* and QC696754 for *C. nigrescens*.

## References

[1] The World Flora Online. http://www.worldfloraonline.org. Accessed Sep 2022 (2022).

[2] Jalas, J., Wyse Jackson, M. B., Sell, P. D. & Whitehead, F. H. *Cerastium* L. *Flora Europaea*. 2nd Ed. Vol. 1 (ed. Tutin, T. G. *et al*.). 164–175. (Cambridge University Press, 1993).

[3] Dequan, L. & Morton, J. *Cerastium* L. *Flora of China*. Vol. 6 (*Caryophyllaceae Through Lardizabalaceae*) (ed. Wu, Z. & Raven, P. H). (Science Press/Missouri Botanical Garden Press, 2001).

[4] Quiroga MP, Premoli AC, Ezcurra C (2002). Morphological and isozyme variation in *Cerastium arvense* (Caryophyllaceae) in the southern Andes. Can. J. Bot..

[5] Nyberg Berglund AB, Saura A, Westerbergh A (2006). Electrophoretic evidence for disomic inheritance and allopolyploid origin of the octoploid *Cerastium alpinum* (Caryophyllaceae). J. Hered..

[6] Hagen AR, Giese H, Brochmann C (2001). Trans-Atlantic dispersal and phylogeography of *Cerastium arcticum* (Caryophyllaceae) inferred from RAPD and SCAR markers. Am. J. Bot..

[7] Caković D, Stešević D, Schönswetter P, Frajman B (2018). Long neglected diversity in the accursed mountains of northern Albania: *Cerastium hekuravense* is genetically and morphologically divergent from *C. dinaricum*. Plant Syst. Evol..

[8] Niketić M, Đurović SZ, Tomović G, Schönswetter P, Frajman B (2022). Diversification within ploidy-variable Balkan endemic *Cerastium decalvans* (Caryophyllaceae) reconstructed based on genetic, morphological and ecological evidence. Bot. J. Linn. Soc..

[9] Milarska SE, Androsiuk P, Bednarek PT, Larson K, Giełwanowska I (2023). Genetic variation of *Cerastium alpinum* L. from Babia Góra, a critically endangered species in Poland. J. Appl. Genet..

[10] Brysting AK, Borgen L (2000). Isozyme analysis of the *Cerastium alpinum*-*C. arcticum* complex (Caryophyllaceae) supports a splitting of *C. arcticum* Lange. Plant Syst. Evol..

[11] Hagen AR (2002). The arctic-alpine polyploids *Cerastium alpinum* and *C. nigrescens* (Caryophyllaceae) in a sympatric situation: Breakdown of species integrity?. Plant Syst. Evol..

[12] Tolmachev A (1930). Die Gattung *Cerastium* in der Flora von Spitzbergen. Skr. Svalb. Ishavet.

[13] Scheen AC (2004). Northern hemisphere biogeography of *Cerastium* (Caryophyllaceae): Insights from phylogenetic analysis of noncoding plastid nucleotide sequences. Am. J. Bot..

[14] Hultén E (1956). The *Cerastium alpinum* complex. A case of world-wide introgressive hybridization. Svensk Bot. Tidskr..

[15] Böcher TW (1977). *Cerastium alpinum* and *C. arcticum*, a mature polyploid complex. Bot. Not..

[16] Rønning OI (1996). The Flora of Svalbard.

[17] Elven, R. & Elvebakk, A. Part 1. Vascular plants. In *A Catalogue of Svalbard Plants, Fungi, Algae, and Cyanobacteria* (eds. Elvebakk, A. & Prestrud, P.). Vol. 198. 9–55 (Norsk Polarinst. Skr., 1996).

[18] Brysting, A. K. & Hagen, A. Species in polyploid complexes? The *Cerastium alpinum*-*C*. *arcticum* complex. *Det Norske Videnskaps Akademi. I. Mat. Nat. Kl. Avh. Ny Serie* **38**, 183–190 (1999).

[19] Brysting AK, Elven R (2000). The *Cerastium alpinum*–*C. arcticum* complex (Caryophyllaceae): Numerical analyses of morphological variation and taxonomical revision of* C. arcticum* Lange. Taxon.

[20] Parusel, J. *Cerastium alpinum* L. s.s. Rogownica alpejska. In *Polska Czerwona Księga Roślin* (ed. Kaźmierczakowa, R. & Zarzycki, K.). 97–99 (Instytut Botaniki im. W. Szafera PAN, Instytut Ochrony Przyrody PAN, 2001).

[21] Parusel, J. *Cerastium alpinum* Rogownica alpejska. In *Polska Czerwona Księga Roślin. Paprotniki i Rośliny Kwiatowe* (eds. Kaźmierczakowa, R., Zarzycki, K. & Mirek, Z.). 121–123 (Instytut Ochrony Przyrody PAN, 2014).

[22] Jansen RK (2007). Analysis of 81 genes from 64 plastid genomes resolves relationships in angiosperms and identifies genome-scale evolutionary patterns. Proc. Natl. Acad. Sci. USA.

[23] Bortiri E, Coleman-Derr D, Lazo G, Anderson O, Gu Y (2008). The complete chloroplast genome sequence of *Brachypodium distachyon*: Sequence comparison and phylogenetic analysis of eight grass plastomes. BMC Res. Notes.

[24] Bock R (2007). Plastid biotechnology: prospects for herbicide and insect resistance, metabolic engineering and molecular farming. Curr. Opin. Biotechnol..

[25] Kress WJ, Wurdack KJ, Zimmer EA, Weigt LA, Janzen DH (2005). Use of DNA barcodes to identify flowering plants. Proc. Natl. Acad. Sci. USA.

[26] Chase MW (2007). A proposal for standardized protocol to barcode all land plants. Taxon.

[27] Raman G, Park S (2016). The complete chloroplast genome sequence of *Ampelopsis*: Gene organization, comparative analysis, and phylogenetic relationships to other angiosperms. Front. Plant Sci..

[28] Zhai Y (2023). Phylogenomics, phylogeography and germplasms authentication of the *Rheum palmatum* complex based on complete chloroplast genomes. J. Plant. Res..

[29] Parks M, Cronn R, Liston A (2009). Increasing phylogenetic resolution at low taxonomic levels using massively parallel sequencing of chloroplast genomes. BMC Biol..

[30] Ohyama K (1986). Chloroplast gene organization deduced from complete sequence of liverwort *Marchantia polymorpha* chloroplast DNA. Nature.

[31] Daniell H, Lin CS, Yu M, Chang WJ (2016). Chloroplast genomes: Diversity, evolution, and applications in genetic engineering. Genome Biol..

[32] Gitzendanner MA, Soltis PS, Wong GK, Ruhfel BR, Soltis DE (2018). Plastid phylogenomic analysis of green plants: A billion years of evolutionary history. Am. J. Bot..

[33] Zhai W (2019). Chloroplast genomic data provide new and robust insights into the phylogeny and evolution of the Ranunculaceae. Mol. Phylogenet. Evol..

[34] Dong W, Xu C, Cheng T, Lin K, Zhou S (2013). Sequencing angiosperm plastid genomes made easy: A complete set of universal primers and a case study on the phylogeny of Saxifragales. Genome Biol. Evol..

[35] Zhu A, Guo W, Gupta S, Fan W, Mower JP (2016). Evolutionary dynamics of the plastid inverted repeat: The effects of expansion, contraction, and loss on substitution rates. New Phytol..

[36] Reith M, Munholland J (1995). Complete nucleotide sequence of the *Porphyra purpurea* chloroplast genome. Plant Mol. Biol. Rep..

[37] Wolfe KH (1988). The site of deletion of the inverted repeat in pea chloroplast DNA contains duplicated gene fragments. Curr. Genet..

[38] Kim KJ, Lee HL (2004). Complete chloroplast genome sequences from Korean ginseng (*Panax schinseng* Nees) and comparative analysis of sequence evolution among 17 vascular plants. DNA Res..

[39] Goulding SE, Olmstead RG, Morden CW, Wolfe KH (1996). Ebb and flow of the chloroplast inverted repeat. Mol. Gen. Genet..

[40] Goremykin VV, Hirsch-Ernst KI, Wölfl S, Hellwig FH (2003). Analysis of the *Amborella trichopoda* chloroplast genome sequence suggests that *Amborella* is not a basal angiosperm. Mol. Biol. Evol..

[41] Hupfer H (2000). Complete nucleotide sequence of the *Oenothera elata* plastid chromosome, representing plastome I of the five distinguishable *Euoenothera* plastomes. Mol. Gen. Genet..

[42] Palmer JD (1983). Chloroplast DNA exists in two orientations. Nature.

[43] Cattolico RA (2008). Chloroplast genome sequencing analysis of *Heterosigma akashiwo* CCMP452 (West Atlantic) and NIES293 (West Pacific) strains. BMC Genom..

[44] Walker JF, Zanis MJ, Emery NC (2014). Comparative analysis of complete chloroplast genome sequence and inversion variation in *Lasthenia burkei* (Madieae, Asteraceae). Am. J. Bot..

[45] Wang M, Cui L, Feng K, Deng P (2015). Comparative analysis of Asteraceae chloroplast genomes: Structural organization, RNA editing and evolution. Plant Mol. Biol. Rep..

[46] Millen RS (2001). Many parallel losses of *infA* from chloroplast DNA during angiosperm evolution with multiple independent transfers to the nucleus. Plant Cell..

[47] Shinozaki K (1986). The complete nucleotide sequence of the tobacco chloroplast genome: Its gene organization and expression. EMBO J..

[48] Sato S, Nakamura Y, Kaneko T, Asamizu E, Tabata S (1999). Complete structure of the chloroplast genome of *Arabidopsis thaliana*. DNA Res..

[49] Scobeyeva VA (2021). Gene loss, pseudogenization in plastomes of genus *Allium* (Amaryllidaceae), and putative selection for adaptation to environmental conditions. Front. Genet..

[50] Raman G, Park S (2015). Analysis of the complete chloroplast genome of a medicinal plant, *Dianthus*
*superbus* var. *longicalyncinus*, from a comparative genomics perspective. PLOS ONE.

[51] Ogihara Y, Terachi T, Sasakuma T (1991). Molecular analysis of the hot spot region related to length mutations in wheat chloroplast DNAs. I. Nucleotide divergence of genes and intergenic spacer regions located in the hot spot region. Genetics.

[52] Lencina F (2019). The *rpl23* gene and pseudogene are hotspots of illegitimate recombination in barley chloroplast mutator seedlings. Sci. Rep..

[53] Skuza L (2023). Molecular structure, comparative and phylogenetic analysis of the complete chloroplast genome sequences of weedy rye *Secale*
*cereale* ssp. *segetale*. Sci. Rep..

[54] Li G, Tembrock LR, Wu Z, Liu F (2019). Complete chloroplast genome of carnation (Caryophyllaceae: *Dianthus*
*caryophyllus* L.). Mitochondrial DNA Part B.

[55] Ling LZ (2020). Characterization of the complete chloroplast genome and phylogenetic analysis of *Silene*
*jenisseensis* (Caryophyllaceae). Mitochondrial DNA Part B.

[56] Kim Y, Heo KI, Lee S, Park J (2019). The complete chloroplast genome sequence of *Pseudostellaria*
*palibiniana* (Takeda) Ohwi (Caryophyllaceae). Mitochondrial DNA Part B.

[57] Kim J, Park J (2019). The complete chloroplast genome sequence of the *Pseudostellaria*
*okamotoi* Ohwi (Caryophyllaceae). Mitochondrial DNA Part B.

[58] Kim Y, Xi H, Park J (2019). The complete chloroplast genome of Prince Ginseng, *Pseudostellaria*
*heterophylla* (Miq.) Pax (Caryophyllaceae). Mitochondrial DNA Part B.

[59] Kim Y, Heo KI, Lee S, Park J (2018). Complete chloroplast genome sequence of the *Pseudostellaria*
*longipedicellata* S. Lee, K. Heo & S. C. Kim (Caryophyllaceae). Mitochondrial DNA Part B.

[60] Yang Z, Zhang Y, Pan L, Fu C (2018). Characterization of the complete chloroplast genome of *Gymnocarpos*
*przewalskii*, an endangered species in China and Mongolia. Conserv. Genet. Resour..

[61] Kang Y (2015). The complete chloroplast genome of Antarctic pearlwort, *Colobanthus*
*quitensis* (Kunth) Bartl. (Caryophyllaceae). Mitochondrial DNA Part A.

[62] Timme RE, Kuehl JV, Boore JL, Jansen RK (2007). A comparative analysis of the *Lactuca* and *Helianthus* (Asteraceae) plastid genomes: Identification of diverged regions and categorization of shared repeats. Am. J. Bot..

[63] Androsiuk P (2018). The complete chloroplast genome of *Colobathus*
*apetalus* (Labill.) Druce: Genome organization and comparison with related species. PeerJ.

[64] Kang JS, Lee BY, Kwak M (2017). The complete chloroplast genome sequences of *Lychnis*
*wilfordii* and *Silene capitata* and comparative analyses with other Caryophyllaceae genomes. PLOS ONE.

[65] Saski C (2005). Complete chloroplast genome sequence of *Glycine*
*max* and comparative analyses with other legume genomes. Plant Mol. Biol..

[66] Lee SB (2006). The complete chloroplast genome sequence of *Gossypium*
*hirsutum*: Organization and phylogenetic relationships to other angiosperms. BMC Genomics.

[67] Jakobsson M, Säll T, Lind-Halldén C, Halldén C (2007). Evolution of chloroplast mononucleotide microsatellites in *Arabidopsis*
*thaliana*. Theor. Appl. Genet..

[68] Hirano R (2011). Propagation management methods have altered the genetic variability of two traditional mango varieties in Myanmar, as revealed by SSR. Plant Genet. Resour. C..

[69] Takahashi D, Sakaguchi S, Isagi Y, Setoguchi H (2018). Comparative chloroplast genomics of series *Sakawanum* in genus *Asarum* (Aristolochiaceae) to develop single nucleotide polymorphisms (SNPs) and simple sequence repeat (SSR) markers. J. For. Res..

[70] Ping J (2021). Molecular evolution and SSRs analysis based on the chloroplast genome of *Callitropsis*
*funebris*. Ecol. Evol..

[71] Androsiuk P (2020). Evolutionary dynamics of the chloroplast genome sequences of six *Colobanthus* species. Sci. Rep..

[72] Liu F (2020). The complete chloroplast genome and characteristics analysis of *Callistemon*
*rigidus* R.Br. Mol. Biol. Rep..

[73] Makalowski W, Boguski MS (1998). Evolutionary parameters of the transcribed mammalian genome: An analysis of 2,820 orthologous rodent and human sequences. Proc. Natl. Acad. Sci. USA.

[74] Newmaster SG, Fazekas AJ, Ragupathy S (2006). DNA barcoding in land plants: evaluation of rbcL in a multigene tiered approach. Can. J. Bot..

[75] Saarela JM, Sokoloff PC, Gillespie LJ, Consaul LL, Bull RD (2013). DNA barcoding the Canadian Arctic Flora: Core plastid barcodes (*rbcL* + *matK*) for 490 vascular plant species. PLOS ONE.

[76] Wang Y (2021). Chloroplast genome variation and phylogenetic relationships of *Atractylodes* species. BMC Genomics.

[77] Choi K, Hwang Y, Hong JK (2022). Comparative chloroplast genomics and phylogenetic analysis of *Persicaria*
*amphibia* (Polygonaceae). Diversity.

[78] Alqahtani AA, Jansen RK (2021). The evolutionary fate of *rpl32* and *rps16* losses in the *Euphorbia*
*schimperi* (Euphorbiaceae) plastome. Sci. Rep..

[79] Nei M, Kumar S (2000). Molecular Evolution and Phylogenetics.

[80] Yang Z, Bielawski JP (2000). Statistical methods for detecting molecular adaptation. Trends Ecol. Evol..

[81] Fu PC, Zhang YZ, Geng HM, Chen SL (2016). The complete chloroplast genome sequence of *Gentiana*
*lawrencei* var. *farreri* (Gentianaceae) and comparative analysis with its congeneric species. PeerJ.

[82] Chen Q, Wu X, Zhang D (2020). Comparison of the abilities of universal, super, and specific DNA barcodes to discriminate among the original species of *Fritillariae*
*cirrhosae* bulbus and its adulterants. PLOS ONE.

[83] Chen C, Xia X, Peng J, Wang D (2022). Comparative analyses of six complete chloroplast genomes from the genus *Cupressus* and *Juniperus* (Cupressaceae). Gene.

[84] Yamori W, Shikanai T (2016). Physiological functions of cyclic electron transport around photosystem I in sustaining photosynthesis and plant growth. Annu. Rev. Plant Biol..

[85] Rumeau D, Peltier G, Cournac L (2007). Chlororespiration and cyclic electron flow around PSI during photosynthesis and plant stress response. Plant Cell Environ..

[86] Piot A, Hackel J, Christin PA, Besnard G (2018). One-third of the plastid genes evolved under positive selection in PACMAD grasses. Planta.

[87] Yang L (2022). Analysis of complete chloroplast genome sequences and insight into the phylogenetic relationships of *Ferula* L.. BMC Genomics.

[88] Yao G (2020). Phylogenetic estimation and morphological evolution of Alsineae (Caryophyllaceae) shed new insight into the taxonomic status of the genus *Pseudocerastium*. Plant Divers..

[89] Sánchez-del Pino I (2020). High phylogeographic and genetic diversity of *Tidestromia*
*lanuginosa* supports full-glacial refugia for arid-adapted plants in southern and central Coahuila, Mexico. Am. J. Bot..

[90] Moonlight PW (2018). Dividing and conquering the fastest-growing genus: Towards a natural sectional classification of the mega-diverse genus *Begonia* (Begoniaceae). Taxon.

[91] Steinhauser S, Beckert S, Capesius I, Malek O, Knoop V (1999). Plant mitochondrial RNA editing. J. Mol. Evol..

[92] Knoop V (2011). When you can’t trust the DNA: RNA editing changes transcript sequences. Cell. Mol. Life Sci..

[93] Tillich M (2005). Editing of plastid RNA in *Arabidopsis thaliana* ecotypes. Plant J..

[94] Chen H, Deng L, Jiang Y, Lu P, Yu J (2011). RNA editing sites exist in protein-coding genes in the chloroplast genome of *Cycas*
*taitungensis*. J. Integr. Plant Biol..

[95] Takenaka M, Zehrmann A, Verbitskiy D, Härtel B, Brennicke A (2013). RNA editing in plants and its evolution. Annu. Rev. Genet..

[96] Benne R (1986). Major transcript of the frame shifted *coxII* gene from trypanosome mitochondria contains four nucleotides that are not encoded in the DNA. Cell.

[97] Covello PS, Gray MW (1989). RNA editing in plant mitochondria. Nature.

[98] Hoch B, Maier RM, Appel K, Igloi GL, Kossel H (1991). Editing of a chloroplast mRNA by creation of an initiation codon. Nature.

[99] Sasaki T, Yukawa Y, Miyamoto T, Obokata J, Sugiura M (2003). Identification of RNA editing sites in chloroplast transcripts from the maternal and paternal progenitors of tobacco (*Nicotiana*
*tabacum*): Comparative analysis shows the involvement of distinct trans-factors for *ndhB* editing. Mol. Biol. Evol..

[100] Corneille S, Lutz K, Maliga P (2000). Conservation of RNA editing between rice and maize plastids: Are most editing events dispensable?. Mol. Gen. Genet..

[101] Miyamoto T, Obokata J, Sugiura M (2002). Recognition of RNA editing sites is directed by unique proteins in chloroplasts: Biochemical identification of *cis*-acting elements and *trans*-acting factors involved in RNA editing in tobacco and pea chloroplasts. Mol. Cell. Biol..

[102] Daniell H (2008). The complete nucleotide sequence of the cassava (*Manihot*
*esculenta*) chloroplast genome and the evolution of *atpF* in Malpighiales: RNA editing and multiple losses of a group II intron. Theor. Appl. Genet..

[103] Germain A, Hotto AM, Barkan A, Stern DB (2013). RNA processing and decay in plastids. Wiley Interdiscip. Rev. RNA.

[104] Ruchika OC, Sakari M, Tsukahara T (2021). Genome-wide identification of U-To-C RNA editing events for nuclear genes in *Arabidopsis*
*thaliana*. Cells.

[105] Xue J, Liu Y, Li L, Wang B, Qiu Y (2010). The complete mitochondrial genome sequence of the hornwort *Phaeoceros*
*laevis*: Retention of many ancient pseudogenes and conservative evolution of mitochondrial genomes in hornworts. Curr. Genet..

[106] Grewe F (2011). A unique transcriptome: 1782 positions of RNA editing alter 1406 codon identities in mitochondrial mRNAs of the lycophyte *Isoetes*
*engelmannii*. Nucleic Acids Res..

[107] Knie N, Grewe F, Knoop V (2016). Monilophyte mitochondrial rps1 genes carry a unique group II intron that likely originated from an ancient paralog in rpl2. RNA.

[108] Rabeler RK, Bittrich V (1993). Suprageneric nomenclature in the Caryophyllaceae. Taxon.

[109] Fior S, Karis PO, Casazza G, Minuto L, Sala F (2006). Molecular phylogeny of the Caryophyllaceae (Caryophyllales) inferred from chloroplast *matK* and nuclear rDNA ITS sequences. Am. J. Bot..

[110] Harbaugh DT (2010). A new lineage-based tribal classification of the family Caryophyllaceae. Int. J. Pl. Sci..

[111] Willis, J. C. *A Dictionary of the Flowering Plants and Ferns*. 8th Ed. 1246 + 66. (Cambridge University Press, 1973).

[112] Pax, F. & Hoffmann, K. Caryophyllaceae. In *Die Natürlichen Pflanzenfamilien Nebst Ihren Gattungen und Wichtigeren Arten, Insbesondere den Nutzpflanzen 16c*. 2nd Ed. (eds. Engler, A. & Harms, H.). 275–364 (Wilhelm Engelmann, 1934).

[113] Niketić M. *Cerastium* L. In *Flora Srbije*. Vol. 2. 2nd Ed. (ed Stevanović, V.). 270–334 (Srpska Akademija Nauka i Umetnosti, 2012).

[114] Brysting AK, Mathiesen C, Marcussen T (2011). Challenges in polyploid phylogenetic reconstruction: A case story from the Arctic-alpine *Cerastium*
*alpinum* complex. Taxon.

[115] Brysting AK (2008). Chromosome number variation in the polyploid *Cerastium*
*alpinum*-*C. arcticum* complex (Caryophyllaceae). Nord. J. Bot..

[116] Greenberg AK, Donoghue MJ (2011). Molecular systematics and character evolution in Caryophyllaceae. Taxon.

[117] Pfeil BE, Schlueter JA, Shoemaker RC, Doyle JJ (2005). Placing paleopolyploidy in relation to taxon divergence: A phylogenetic analysis in legumes using 39 gene families. Syst. Biol..

[118] Xue B (2023). Phylogenetic analysis and temporal diversification of the tribe Alsineae (Caryophyllaceae) with the description of three new genera, *Hesperostellaria*, *Reniostellaria* and *Torreyostellaria*. Front. Plant Sci..

[119] Frajman B, Eggens F, Oxelman B (2009). Hybrid origins and homoploid reticulate evolution within *Heliosperma* (Sileneae, Caryophyllaceae)—A multigene phylogenetic approach with relative dating. Syst. Biol..

[120] Vít P, Wolfová K, Urfus T, Tájek P, Suda J (2014). Interspecific hybridization between rare and common plant congeners inferred from genome size data: assessing the threat to the Czech serpentine endemic *Cerastium*
*alsinifolium*. Preslia.

[121] Nosil P (2012). Ecological Speciation.

[122] Tonti-Filippini J, Nevill PG, Dixon K, Small I (2017). What can we do with 1000 plastid genomes?. Plant J..

[123] McKain MR, Johnson MG, Uribe-Convers S, Eaton D, Yang Y (2018). Practical considerations for plant phylogenomics. Appl. Plant Sci..

[124] Kearse M (2012). Geneious basic: An integrated and extendable desktop software platform for the organization and analysis of sequence data. Bioinformatics.

[125] Dong X, Stothard P, Forsythe IJ, Wishart DS (2004). PlasMapper: A web server for drawing and auto-annotating plasmid maps. Nucleic Acids Res..

[126] Lohse M, Drechsel O, Bock R (2007). OrganellarGenomeDRAW (OGDRAW)—A tool for the easy generation of high-quality custom graphical maps of plastid and mitochondrial genomes. Curr. Genet..

[127] Jin JJ (2020). GetOrganelle: A fast and versatile toolkit for accurate de novo assembly of organelle genomes. Genome Biol..

[128] Kurtz S (2001). REPuter: The manifold applications of repeat analysis on a genomic scale. Nucleic Acids Res..

[129] Mayer, C. *2006–2010. Phobos 3.3.11. A Tandem Repeat Search Program. 2006–2010.*

[130] Sablok G (2015). ChloroMitoSSRDB 2.00: More genomes, more repeats, unifying SSRs search patterns and on-the-fly repeat detection. Database.

[131] Darling ACE, Mau B, Blattner FR, Perna NT (2004). Mauve: Multiple alignment of conserved genomic sequence with rearrangements. Genome Res..

[132] Katoh K, Standley DM (2013). MAFFT multiple sequence alignment software version 7: Improvements in performance and usability. Mol. Biol. Evol..

[133] Rozas J (2017). DnaSP 6: DNA sequence polymorphism analysis of large datasets. Mol. Biol. Evol..

[134] Krzywinski M (2009). Circos: An information aesthetic for comparative genomics. Genome Res..

[135] Lenz H, Hein A, Knoop V (2018). Plant organelle RNA editing and its specificity factors: Enhancements of analyses and new database features in PREPACT 3.0. BMC Bioinform..

[136] Kumar S, Stecher G, Tamura K (2016). MEGA7: Molecular evolutionary genetics analysis version 7.0 for bigger datasets. Mol. Biol. Evol..

[137] Huelsenbeck JP, Ronquist F (2001). MRBAYES: Bayesian inference of phylogenetic trees. Bioinformatics.

[138] Ronquist F, Huelsenbeck JP (2003). MrBayes 3: Bayesian phylogenetic inference under mixed models. Bioinformatics.

[139] Kumar S, Stecher G, Suleski M, Hedges SB (2017). TimeTree: A resource for timelines, timetrees, and divergence times. Mol. Biol. Evol..

