# Supplementary material for: Complete chloroplast genomes of Cerastium alpinum, C. arcticum and C. nigrescens: genome structures, comparative and phylogenetic analysis
Source: Sci Rep. 2023 Oct 31;13:18774. doi: 10.1038/s41598-023-46017-y (PMC10618263; doi:10.1038/s41598-023-46017-y)
Supplement: Supplementary file 2 — Supplementary Figure S2. [file 41598_2023_46017_MOESM2_ESM.pdf]

Complete chloroplast genomes of *Cerastium alpinum*, *C. arcticum* and *C. nigrescens*: genome structures, comparative and phylogenetic analysis

Sylwia E. Milarska[1], Piotr Androsiuk[1]*, Łukasz Paukszto[2], Jan P. Jastrzębski[1], Mateusz Maździarz[2], Keith Larson[3] and Irena Giełwanowska[1]

[1.] Department of Plant Physiology, Genetics and Biotechnology, Faculty of Biology and Biotechnology, University of Warmia and Mazury in Olsztyn, ul. M. Oczapowskiego 1A, 10-719 Olsztyn, Poland;

[2] Department of Botany and Nature Protection, Faculty of Biology and Biotechnology, University of Warmia and Mazury in Olsztyn, Pl. Łódzki 1, 10-721 Olsztyn, Poland;

[3.] Climate Impacts Research Centre, Department of Ecology and Environmental Sciences, Umeå University, 901 87, Umeå, Sweden.

* corresponding author – piotr.androsiuk@uwm.edu.pl

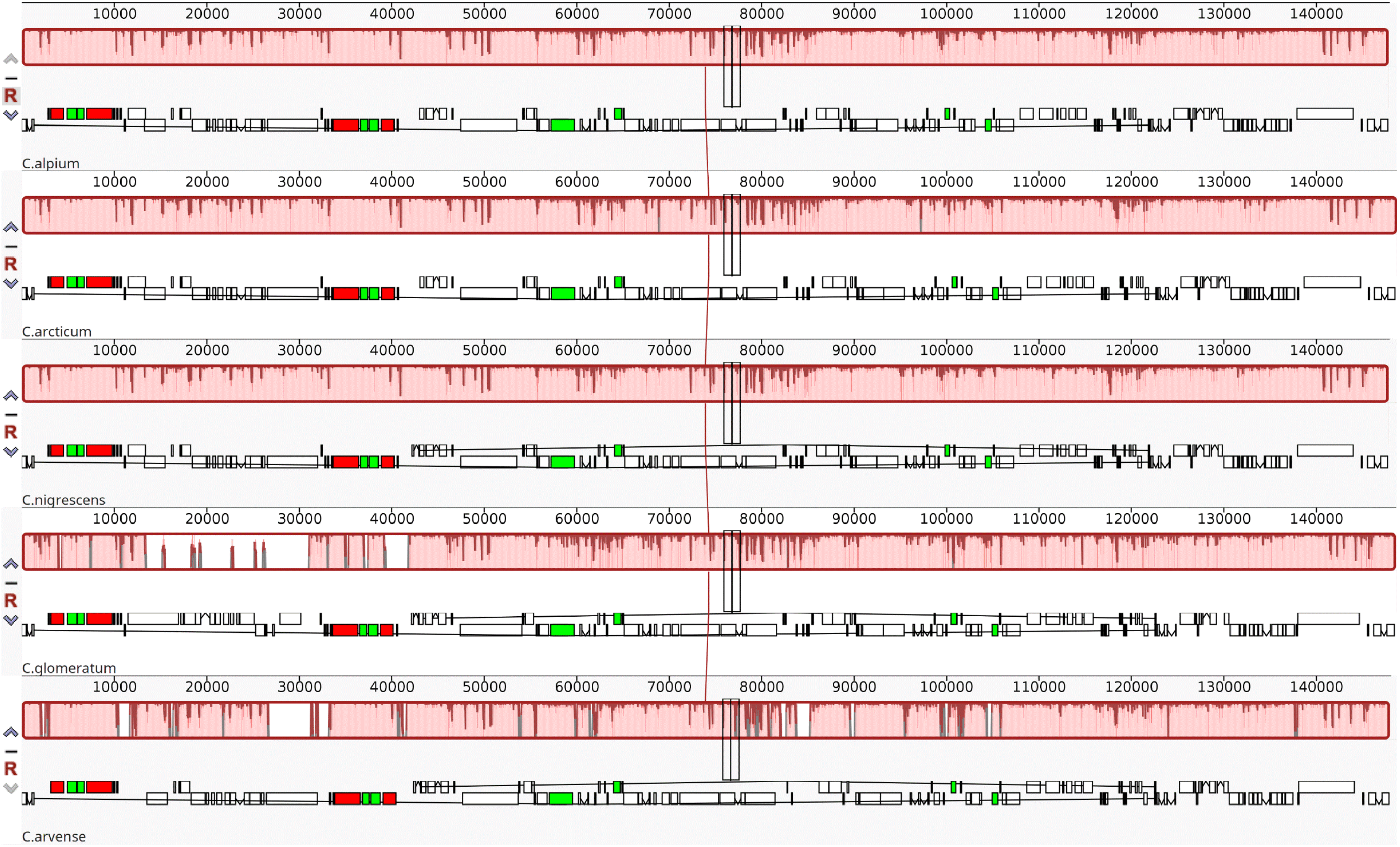

Figure S2. A MAUVE alignment of *C. alpinum C. arcticum*, *C. nigrescens. C. glomeratum*, and *C. arvense* chloroplast genomes. Within each of the alignment, local collinear blocks are represented by blocks of the same color connected by lines.
