# Supplementary material for: Complete chloroplast genomes of Cerastium alpinum, C. arcticum and C. nigrescens: genome structures, comparative and phylogenetic analysis
Source: Sci Rep. 2023 Oct 31;13:18774. doi: 10.1038/s41598-023-46017-y (PMC10618263; doi:10.1038/s41598-023-46017-y)
Supplement: Supplementary file 6 — Supplementary Legends. [file 41598_2023_46017_MOESM6_ESM.pdf]

Supplementary file legend:

Supplementary Table S1 Codon usage in all protein-coding genes for three *Cerastium* species.

Supplementary Table S2A. List of repeated sequences in the chloroplast genome of *Cerastium alpinum.* **Table S2B.** List of repeated sequences in the chloroplast genome of *Cerastium arcticum.* **Table S2C.** List of repeated sequences in the chloroplast genome of *Cerastium nigrescens.* **Table S2D.** List of repeated sequences in the chloroplast genome of *Cerastium glomeratum.*

Supplementary Table S3A. Distribution of SSR in the *Cerastium alpinum* cp genome; **Table S3B.** Distribution of SSR in the *Cerastium arcticum* cp genome. **Table S3C.** Distribution of SSR in the *Cerastium nigrescens* cp genome. **Table S3D.** Distribution of SSR in the *Cerastium glomeratum* cp genome.

Supplementary Table S4. Ks and Ka values of *C. glomeratum* chloroplast genome vs. other representatives of genus *Cerastium.*

Supplementary Table S5A. Predicted potential RNA editing sites in the protein-coding genes of *Cerastium alpinum* chloroplast genome. **Table S5B.** Predicted potential RNA editing sites in the protein-coding genes of *Cerastium arcticum* chloroplast genome. **Table S5C.** Predicted potential RNA editing sites in the protein-coding genes of *Cerastium nigrescens* chloroplast genome. **Table S5D.** Predicted potential RNA editing sites in the protein-coding genes of *Cerastium glomeratum* chloroplast genome. **Table S5E.** Predicted potential RNA editing sites in the protein-coding genes of *Cerastium arvense* chloroplast genome.

Supplementary Figure S1. The chloroplast genome coverage depth of *Cerastium alpinum* (**A**), *Cerastium arcticum* (**B**) and *Cerastium nigrescens* (**C**).

Supplementary Figure S2. A MAUVE alignment of *C. alpinum C. arcticum*, *C. nigrescens. C. glomeratum,* and *C. arvense* chloroplast genomes. Within each of the alignment, local collinear blocks are represented by blocks of the same color connected by lines.

Supplementary Figure S3. *Cerastium alpinum*

Supplementary Figure S4. *Cerastium arcticum*

Supplementary Figure S5. *Cerastium nigrescens*
