# Supplementary material for: Complete chloroplast genomes of Cerastium alpinum, C. arcticum and C. nigrescens: genome structures, comparative and phylogenetic analysis
Source: Sci Rep. 2023 Oct 31;13:18774. doi: 10.1038/s41598-023-46017-y (PMC10618263; doi:10.1038/s41598-023-46017-y)
Supplement: Supplementary file 8 — Supplementary Table S2. [file 41598_2023_46017_MOESM8_ESM.pdf]

Complete chloroplast genomes of *Cerastium alpinum, C. arcticum* and *C. nigrescens*: genome structures, comparative and phylogenetic analysis

Sylwia E. Milarska[1], Piotr Androsiuk[1]*, Łukasz Paukszto[2], Jan P. Jastrzębski[1], Mateusz Maździarz[2], Keith Larson[3] and Irena Giełwanowska[1]

[1.] Department of Plant Physiology, Genetics and Biotechnology, Faculty of Biology and Biotechnology, University of Warmia and Mazury in Olsztyn, ul. M. Oczapowskiego 1A, 10-719 Olsztyn, Poland;

[2] Department of Botany and Nature Protection, Faculty of Biology and Biotechnology, University of Warmia and Mazury in Olsztyn, Pl. Łódzki 1, 10-721 Olsztyn, Poland;

[3.] Climate Impacts Research Centre, Department of Ecology and Environmental Sciences, Umeå University, 901 87, Umeå, Sweden.

* corresponding author – piotr.androsiuk@uwm.edu.pl

Table S2A. List of repeated sequences in the chloroplast genome of *Cerastium alpinum*

Repeat length (bp)	Strat site of repeat A	Repeat A location	Repeat A region	Strat site of repeat B	Repeat B location	Repeat B region	Repeat type
30	63,333	*trnS-GCU*	LSC	97,992	*trnS-GGA*	LSC	P
30	58,549	*matK*	LSC	58,549	*matK*	LSC	P
30	88,609	*trnS-UGA*	LSC	97,992	*trnS-GGA*	LSC	P
30	91,702	*psaB*	LSC	93,926	*psaA*	LSC	F
30	100,208	*trnL-UAA* (intron)	LSC	100,208	*trnL-UAA* (intron)	LSC	P
30	113,132	IGS (*ycf4-cemA*)	LSC	113,132	IGS (*ycf4-cemA*)	LSC	R
30	1,460	IGS (*rps12-rrn16*)	IR	96,827	*ycf3* (intron 1)	LSC	F
30	63,333	*trnS-GCU*	LSC	88,609	*trnS-UGA*	LSC	F
30	64,936	*trnG-UCC*	LSC	89,510	IGS (*psbZ-rps14*)	LSC	F
31	139,955	*ycf2*	IR	139,976	*ycf2*	IR	F
32	55,946	IGS (*trnH-GUG-psbA*)	LSC	55,984	IGS (*trnH-GUG-psbA*)	LSC	P
34	65,218	IGS (*trnR-UCU-atpA*)	LSC	67,647	*atpF* (intron)	LSC	P
35	95,382	IGS (*psaA-ycf3*)	LSC	112,340	IGS (*psaI-ycf4*)	LSC	P
36	82,993	IGS (*petN-psbM*)	LSC	82,993	IGS (*petN-psbM*)	LSC	P
37	23,345	*ndhA* (intron)	SSC	96,815	*ycf3* (intron 1)	LSC	F
39	1,446	IGS (*rps12-rrn16*)	IR	23,343	*ndhA* (intron)	SSC	F
39	23,343	*ndhA* (intron)	SSC	42,182	IGS (*rrn16-rps12*)	IR	P
39	1,448	IGS (*rps12-rrn16*)	IR	96,815	*ycf3* (intron 1)	LSC	F
46	20,047	IGS (*ndhD-psaC*)	SSC	20,047	IGS (*ndhD-psaC*)	SSC	P
73	95,310	IGS (*psaA-ycf3*)	LSC	112,375	IGS (*psaI-ycf4*)	LSC	P

IGS (*ycf4-cemA*) means spacer between *ycf4* and *cemA*, P means palindromic match, F means forward (direst) match and R means reverse match

Table S2B. List of repeated sequences in the chloroplast genome of *Cerastium arcticum*

Repeat length (bp)	Strat site of repeat A	Repeat A location	Repeat A region	Strat site of repeat B	Repeat B location	Repeat B region	Repeat type
30	63,359	*trnS-GCU*	LSC	98,903	*trnS-GGA*	LSC	P
30	58,564	*matK*	LSC	58,564	*matK*	LSC	P
30	89,326	*trnS-UGA*	LSC	98,903	*trnS-GGA*	LSC	P
30	92,419	*psaB*	LSC	94,643	*psaA*	LSC	F
30	113,900	IGS (*ycf4-cemA*)	LSC	113,900	IGS (*ycf4-cemA*)	LSC	R
30	1,451	IGS (*rps12-rrn16*)	IR	97,732	*ycf3* (intron 1)	LSC	F
30	63,359	*trnS-GCU*	LSC	89,326	*trnS-UGA*	LSC	F
30	64,942	*trnG-UCC*	LSC	90,227	IGS (*psbZ-rps14*)	LSC	F
31	65,242	IGS (*trnR-UCU-atpA*)	LSC	67,677	*atpF* (intron)	LSC	P
31	140,727	*ycf2*	IR	140,748	*ycf2*	IR	F
31	97,369	*ycf3* (intron 2)	LSC	97,383	*ycf3* (intron 2)	LSC	F
33	55,959	IGS (*trnH-GUG-psbA*)	LSC	55,998	IGS (*trnH-GUG-psbA*)	LSC	P
34	100,973	*trnL-UAA* (intron)	LSC	100,977	*trnL-UAA* (intron)	LSC	P
36	83,701	IGS (*petN-psbM*)	LSC	83,701	IGS (*petN-psbM*)	LSC	P
37	23,356	*ndhA* (intron)	SSC	97,720	*ycf3* (intron 1)	LSC	F
39	1,437	IGS (*rps12-rrn16*)	IR	23,354	*ndhA* (intron)	SSC	F
39	23,354	*ndhA* (intron)	SSC	42,199	IGS (*rrn16-rps12*)	IR	P
39	1,439	IGS (*rps12-rrn16*)	IR	97,720	*ycf3* (intron 1)	LSC	F
43	97,333	*ycf3* (intron 2)	LSC	97,360	*ycf3* (intron 2)	LSC	F
46	20,055	IGS (*ndhD-psaC*)	SSC	20,055	IGS (*ndhD-psaC*)	SSC	P
61	68,941	IGS (*atpH-atpI*)	LSC	69,002	IGS (*atpH-atpI*)	LSC	F
72	96,199	IGS (*psaA-ycf3*)	LSC	113,109	IGS (*psaI-ycf4*)	LSC	P
162	96,109	IGS (*psaA-ycf3*)	LSC	113,109	IGS (*psaI-ycf4*)	LSC	P

IGS (*ycf4-cemA*) means spacer between *ycf4* and *cemA*, P means palindromic match, F means forward (direst) match and R means reverse match

Table S2C. List of repeated sequences in the chloroplast genome of *Cerastium nigrescens*

Repeat length (bp)	Strat site of repeat A	Repeat A location	Repeat A region	Strat site of repeat B	Repeat B location	Repeat B region	Repeat type
30	63,336	*trnS-GCU*	LSC	98,004	*trnS-GGA*	LSC	P
30	585,48	*matK*	LSC	58,548	*matK*	LSC	P
30	88,612	*trnS-UGA*	LSC	98,004	*trnS-GGA*	LSC	P
30	91,705	*psaB*	LSC	93,929	*psaA*	LSC	F
30	100,215	*trnL-UAA* (intron)	LSC	100,215	*trnL-UAA* (intron)	LSC	P
30	113,134	IGS (*ycf4-cemA*)	LSC	113,134	IGS (*ycf4-cemA*)	LSC	R
30	1,460	IGS (*rps12-rrn16*)	IR	96,839	*ycf3* (intron 1)	LSC	F
30	63,336	*trnS-GCU*	LSC	88,612	*trnS-UGA*	LSC	F
30	64,939	*trnG-UCC*	LSC	89,513	IGS (*psbZ-rps14*)	LSC	F
31	139,950	*ycf2*	IR	139,971	*ycf2*	IR	F
32	55,945	IGS (*trnH-GUG-psbA*)	LSC	55,983	IGS (*trnH-GUG-psbA*)	LSC	P
34	65,221	IGS (*trnR-UCU-atpA*)	LSC	67,650	*atpF* (intron)	LSC	P
35	95,394	IGS (*psaA-ycf3*)	LSC	112,342	IGS (*psaI-ycf4*)	LSC	P
36	82,996	IGS (*petN-psbM*)	LSC	82,996	IGS (*petN-psbM*)	LSC	P
37	23,345	*ndhA* (intron)	SSC	96,827	*ycf3* (intron 1)	LSC	F
39	1,446	IGS (*rps12-rrn16*)	IR	23,343	*ndhA* (intron)	SSC	F
39	23,343	*ndhA* (intron)	SSC	42,181	IGS (*rrn16-rps12*)	IR	P
39	1,448	IGS (*rps12-rrn16*)	IR	96,827	*ycf3* (intron 1)	LSC	F
46	20,047	IGS (*ndhD-psaC*)	SSC	20,047	IGS (*ndhD-psaC*)	SSC	P
73	95,322	IGS (*psaA-ycf3*)	LSC	112,377	IGS (*psaI-ycf4*)	LSC	P

IGS (*ycf4-cemA*) means spacer between *ycf4* and *cemA*, P means palindromic match, F means forward (direst) match and R means reverse match

Table S2D. List of repeated sequences in the chloroplast genome of *Cerastium glomeratum*

Repeat length (bp)	Strat site of repeat A	Repeat A location	Repeat A region	Strat site of repeat B	Repeat B location	Repeat B region	Repeat type
30	63,304	*trnS-GCU*	LSC	98,815	*trnS-GGA*	LSC	P
30	89,290	*trnS-UGA*	LSC	98,815	*trnS-GGA*	LSC	P
30	92,395	*psaB*	LSC	94,619	*psaA*	LSC	F
30	113,867	IGS (*ycf4-cemA*)	LSC	113,867	IGS (*ycf4-cemA*)	LSC	R
30	63,304	*trnS-GCU*	LSC	89,290	*trnS-UGA*	LSC	F
30	64,873	*trnG-UCC*	LSC	90,194	*trnG-GCC*	LSC	F
31	140,629	*ycf2*	IR	140,650	*ycf2*	IR	F
31	100,965	*trnL-UAA* (intron)	LSC	100,965	*trnL-UAA* (intron)	LSC	P
33	55,897	IGS (*trnH-GUG-psbA*)	LSC	55,936	IGS (*trnH-GUG-psbA*)	LSC	P
36	58,506	*matK*	LSC	58,506	*matK*	LSC	P
36	83,674	IGS (*petN-psbM*)	LSC	83,674	IGS (*petN-psbM*)	LSC	P
39	1,446	IGS (*rps12-trnV-GAC*)	IR	20,343	*ndhA* (intron)	SSC	P
39	20,343	*ndhA* (intron)	SSC	42,115	IGS (*trnV-GAC-rps12*)	IR	F
46	23,632	IGS (*psaC-ndhD*)	SSC	23,632	IGS (*psaC-ndhD*)	SSC	P
85	96,157	IGS (*psaA-ycf3*)	LSC	113,077	IGS (*psaI-ycf4*)	LSC	P
170	96,072	IGS (*psaA-ycf3*)	LSC	113,077	IGS (*psaI-ycf4*)	LSC	P

IGS (*ycf4-cemA*) means spacer between *ycf4* and *cemA*, P means palindromic match, F means forward (direst) match and R means reverse match
