# Supplementary material for: Complete chloroplast genomes of Cerastium alpinum, C. arcticum and C. nigrescens: genome structures, comparative and phylogenetic analysis
Source: Sci Rep. 2023 Oct 31;13:18774. doi: 10.1038/s41598-023-46017-y (PMC10618263; doi:10.1038/s41598-023-46017-y)
Supplement: Supplementary file 9 — Supplementary Table S3. [file 41598_2023_46017_MOESM9_ESM.pdf]

# **Complete chloroplast genomes of *Cerastium alpinum*, *C. arcticum* and *C. nigrescens*: genome structures, comparative and phylogenetic analysis**

Sylwia E. Milarska[1], Piotr Androsiuk[1]*, Łukasz Paukszto[2], Jan P. Jastrzębski[1], Mateusz Maździarz[2], Keith Larson[3] and Irena Giełwanowska[1]

[1.] Department of Plant Physiology, Genetics and Biotechnology, Faculty of Biology and Biotechnology, University of Warmia and Mazury in Olsztyn, ul. M. Oczapowskiego 1A, 10-719 Olsztyn, Poland;

[2] Department of Botany and Nature Protection, Faculty of Biology and Biotechnology, University of Warmia and Mazury in Olsztyn, Pl. Łódzki 1, 10-721 Olsztyn, Poland;

[3.] Climate Impacts Research Centre, Department of Ecology and Environmental Sciences, Umeå University, 901 87, Umeå, Sweden.

* corresponding author – piotr.androsiuk@uwm.edu.pl

**Table S3A.** Distribution of SSR in the *Cerastium alpinum* cp genome.

| Type | Repeat unit | Length (bp) | Start | End | Location |
|---|---|---|---|---|---|
| Mononucleotide | A | 12 | 11,878 | 11,889 | *ycf1* |
| | | 13 | 28,119 | 28,131 | *ycf1* |
| | | 12 | 58,788 | 58,799 | *matK* |
| | | 12 | 73,365 | 73,376 | *rpoC2* |
| | | 13 | 83,607 | 83,619 | IGS (*psbM-psbD*) |
| | | 12 | 85,146 | 85,157 | IGS (*psbM-psbD*) |
| | | 12 | 95,444 | 95,455 | IGS (*psaA-ycf3*) |
| | | 12 | 99,608 | 99,619 | IGS (*trnT-UGU-trnL-UAA*) |
| | | 13 | 133,151 | 133,163 | IGS (*rpl14-rpl16*) |
| Trinucleotide | AAT | 13 | 14,074 | 14,086 | *ndhF* |
| | | 12 | 63,563 | 63,574 | IGS (*trnS-GCU-trnG-UCC*) |
| | | 14 | 63,906 | 63,919 | IGS (*trnS-GCU-trnG-UCC*) |
| | | 14 | 97,915 | 97,928 | IGS (*ycf3-trnS-GGA*) |
| | | 17 | 99,389 | 99,405 | IGS (*trnT-UGU-trnL-UAA*) |
| | | 12 | 118,910 | 118,921 | IGS (*trnP-UGG-psaJ*) |
| | | 12 | 120,081 | 120,092 | IGS (*rpl33-rps18*) |
| Tetranucleotide | ACCT | 14 | 8,761 | 8,774 | *rrn23* |
| | AAAT | 12 | 18,720 | 18,731 | *ndhD* |
| | AATT | 12 | 63,461 | 63,472 | IGS (*trnS-GCU-trnG-UCC*) |
| | AATT | 12 | 100,217 | 100,228 | *trnL-UAA* (intron) |
| Pentanucleotide | AATAT | 16 | 99,417 | 99,432 | IGS (*trnT-UGU-trnL-UAA*) |

IGS (*psbM-psbD* ) means spacer between *psbM* and *psbD*

**Table S3B.** Distribution of SSR in the *Cerastium arcticum* cp genome.

| Type | Repeat unit | Length (bp) | Start | End | Location |
|---|---|---|---|---|---|
| Mononucleotide | A | 12 | 11,877 | 11,888 | *ycf1* |
| | | 13 | 28,131 | 28,143 | *ycf1* |
| | | 12 | 58,803 | 58,814 | *matK* |
| | | 12 | 73,460 | 73,471 | *rpoC2* |
| | | 18 | 84,321 | 84,338 | IGS (*psbM-psbD*) |
| | | 12 | 98,695 | 98,706 | IGS (*ycf3-trnS-GGA*) |
| | | 14 | 98,799 | 98,812 | IGS (*ycf3-trnS-GGA*) |
| | | 13 | 100,388 | 100,400 | IGS (*trnT-UGU-trnL-UAA*) |
| | | 12 | 119,927 | 119,938 | IGS (*trnP-UGG-psaJ*) |
| | | 14 | 133,918 | 133,931 | IGS (*rpl14-rpl16*) |
| Trinucleotide | AAT | 13 | 14,073 | 14,085 | *ndhF* |
| | | 12 | 63,567 | 63,578 | IGS (*trnS-GCU-trnG-UCC*) |
| | | 16 | 71,387 | 71,402 | IGS (*rps2-rpoC2*) |
| | | 14 | 98,826 | 98,839 | IGS (*ycf3-trnS-GGA*) |
| | | 17 | 100,304 | 100,320 | IGS (*trnT-UGU-trnL-UAA*) |
| | | 12 | 119,684 | 119,695 | IGS (*trnP-UGG-psaJ*) |
| | | 12 | 120,864 | 120,875 | IGS (*rpl33-rps18*) |
| Tetranucleotide | ACCT | 14 | 8,756 | 8,769 | *rrn23* |
| | AAAT | 12 | 18,728 | 18,739 | *ndhD* |
| | AATT | 12 | 63,487 | 63,498 | IGS (*trnS-GCU-trnG-UCC*) |
| | AGAT | 12 | 101,035 | 101,046 | *trnL-UAA* (intron) |
| | AATT | 12 | 134,509 | 134,520 | *rpl16* (intron) |
| Hexanucleotide | AAATCC | 19 | 29,127 | 29,145 | *ycf1* |

IGS (*psbM-psbD* ) means spacer between *psbM* and *psbD*

**Table S3C.** Distribution of SSR in the *Cerastium nigrescens* cp genome.

| Type | Repeat unit | Length (bp) | Start | End | Location |
|---|---|---|---|---|---|
| Mononucleotide | A | 12 | 11,878 | 11,889 | *ycf1* |
| | | 13 | 28,118 | 28,130 | *ycf1* |
| | | 12 | 58,787 | 58,798 | *matK* |
| | | 12 | 73,368 | 73,379 | *rpoC2* |
| | | 13 | 83,610 | 83,622 | IGS (*psbM-psbD*) |
| | | 12 | 85,149 | 85,160 | IGS (*psbM-psbD*) |
| | | 12 | 99,615 | 99,626 | IGS (*trnT-UGU-trnL-UAA*) |
| | | 13 | 133,146 | 133,158 | IGS (*rpl14-rpl16*) |
| Trinucleotide | AAT | 13 | 14,074 | 14,086 | *ndhF* |
| | | 12 | 63,566 | 63,577 | IGS (*trnS-GCU-trnG-UCC*) |
| | | 15 | 64,281 | 64,295 | *trnG-UCC* (intron) |
| | | 14 | 97,927 | 97,940 | IGS (*ycf3-trnS-GGA*) |
| | | 17 | 99,401 | 99,417 | IGS (*trnT-UGU-trnL-UAA*) |
| | | 12 | 118,915 | 118,926 | IGS (*trnP-UGG-psaJ*) |
| | | 12 | 120,090 | 120,101 | IGS (*rpl33-rps18*) |
| Tetranucleotide | ACCT | 14 | 8,761 | 8,774 | *rrn23* |
| | AAAT | 12 | 18,720 | 18,731 | *ndhD* |
| | AATT | 12 | 63,464 | 63,475 | IGS (*trnS-GCU-trnG-UCC*) |
| | AATT | 12 | 100,224 | 100,235 | *trnL-UAA* (intron) |
| | AAAT | 13 | 119,006 | 119,018 | IGS (*trnP-UGG-psaJ*) |

IGS (*psbM-psbD* ) means spacer between *psbM* and *psbD*

**Table S3D.** Distribution of SSR in the *Cerastium glomeratum* cp genome.

| Type | Repeat unit | Length (bp) | Start | End | Location |
|---|---|---|---|---|---|
| Mononucleotide | A | 12 | 11,891 | 11,902 | *ycf1* |
| | | 13 | 15,556 | 15,568 | *ycf1* |
| | | 12 | 21,753 | 21,764 | IGS (*ndhI-ndhG*) |
| | | 12 | 58,751 | 58,762 | *matK* |
| | | 12 | 62,984 | 62,995 | IGS (*psbK-psbI*) |
| | | 12 | 73,367 | 73,378 | *rpoC2* |
| | | 13 | 98,611 | 98,623 | IGS (*ycf3-trnS-GGA*) |
| | | 12 | 104,764 | 104,775 | IGS (*ndhC-trnV-UAC*) |
| Dinucleotide | AT | 13 | 89,161 | 89,173 | IGS (*psbC-trnS-UGA*) |
| Trinucleotide | AAT | 13 | 29,502 | 29,514 | *ndhF* |
| | | 12 | 63,517 | 63,528 | IGS (*trnS-GCU-trnG-UCC*) |
| | | 13 | 63,653 | 63,665 | IGS (*trnS-GCU-trnG-UCC*) |
| | | 14 | 98,738 | 98,751 | IGS (*ycf3-trnS-GGA*) |
| | | 12 | 120,777 | 120,788 | IGS (*rpl33-rps18*) |
| | | 13 | 145,896 | 145,908 | IGS (*trnL-CAA-ndhB*) |
| Tetranucleotide | ACCT | 14 | 8,775 | 8,788 | *rrn23* |
| | AAAT | 12 | 24,995 | 25,006 | *ndhD* |
| | AATT | 12 | 98,268 | 98,279 | *ycf3* (intron 1) |
| | AATT | 13 | 99,791 | 99,803 | IGS (*rps4-trnT-UGU*) |
| | AAAT | 12 | 110,398 | 110,409 | IGS (*rbcL-accD*) |
| | AAAG | 12 | 118,080 | 118,091 | IGS (*psbE-petL*) |
| | AAAT | 12 | 124,724 | 124,735 | *clpP* (intron 1) |

IGS (*ndhI-ndhG*) means spacer between *ndhI* and *ndhG*
